# Human alveolar macrophages display marked hypo-responsiveness to IFN-γ in both proteomic and gene expression analysis

**DOI:** 10.1371/journal.pone.0295312

**Published:** 2024-02-01

**Authors:** Bonnie A. Thiel, Kathleen C. Lundberg, Daniela Schlatzer, Jessica Jarvela, Qing Li, Rachel Shaw, Scott M. Reba, Shane Fletcher, Sara E. Beckloff, Mark R. Chance, W. Henry Boom, Richard F. Silver, Gurkan Bebek

**Affiliations:** 1 Department of Medicine, Case Western Reserve University and University Hospitals Cleveland Medical Center, Cleveland, Ohio, United States of America; 2 Department of Nutrition, Center for Proteomics and Bioinformatics, Case Western Reserve University School of Medicine, Cleveland, Ohio, United States of America; 3 Division of Pulmonary, Critical Care, and Sleep Medicine, Louis Stokes Cleveland Department of Veterans Affairs Medical Center, Cleveland, Ohio, United States of America; 4 Biobot Analytics, Cambridge, Massachusetts, United States of America; 5 Division of Pulmonary, Critical Care, and Sleep Medicine, University Hospitals Case Medical Center and Case Western Reserve University School of Medicine, Cleveland, Ohio, United States of America; Chittaranjan National Cancer Institute, INDIA

## Abstract

Alveolar macrophages (AM) perform a primary defense mechanism in the lung through phagocytosis of inhaled particles and microorganisms. AM are known to be relatively immunosuppressive consistent with the aim to limit alveolar inflammation and maintain effective gas exchange in the face of these constant challenges. How AM respond to T cell derived cytokine signals, which are critical to the defense against inhaled pathogens, is less well understood. For example, successful containment of *Mycobacterium tuberculosis* (Mtb) in lung macrophages is highly dependent on IFN-γ secreted by Th-1 lymphocytes, however, the proteomic IFN-γ response profile in AM remains mostly unknown. In this study, we measured IFN-γ induced protein abundance changes in human AM and autologous blood monocytes (MN). AM cells were activated by IFN-γ stimulation resulting in STAT1 phosphorylation and production of MIG/CXCL9 chemokine. However, the global proteomic response to IFN-γ in AM was dramatically limited in comparison to that of MN (9 AM vs 89 MN differentially abundant proteins). AM hypo-responsiveness was not explained by reduced JAK-STAT1 signaling nor increased SOCS1 expression. These findings suggest that AM have a tightly regulated response to IFN-γ which may prevent excessive pulmonary inflammation but may also provide a niche for the initial survival and growth of Mtb and other intracellular pathogens in the lung.

## Introduction

Bronchoscopy with bronchoalveolar lavage provides a unique opportunity to study primary tissue macrophages that are relevant to the control of respiratory infections. Recent studies of tissue macrophage development have changed a longstanding paradigm in indicating that alveolar macrophages (AM) are not primarily derived from differentiation of extravasated peripheral blood monocytes (MN) which are themselves derived from myeloid precursors. In contrast, the migration of macrophages to the lung occurs earlier in development, when the fetal liver is the main source of hematopoiesis. This differing ontogeny appears to have a marked impact on the functional qualities of AM vs MN. In the broadest since, AM display an anti-inflammatory phenotype that is critical to balancing the need to respond appropriately to pathogens with an ability to limit local inflammation and thus preserve the gas-exchange function of the lung [1, 2]. In the context of understanding protective immunity to respiratory pathogens, the differences between AM and MN indicate studying primary human AM rather than in vitro models based on cytokine induced differentiation of blood MN.

Our prior studies have largely focused on assessing local immunity to mycobacterium tuberculosis (Mtb) within the human lung. Specifically, we have evaluated individuals with latent tuberculosis infection (LTBI), i.e. infected with this respiratory pathogen and subsequently developed specific cell-mediated immunity that contains but does not eliminate Mtb. Because of the property of memory T cells to preferentially re-circulate to the site at which they were first exposed to a pathogen (ie, “homing”), we have proposed that LTBI my serve as a human model of effective immunity to Mtb. Our studies have indicated that Mtb-responsive CD4+ T cells are greatly enriched within the distal airways compared to peripheral blood; further, IFN-γ -dependent chemokines are a key element of recruitment of additional immune cells to the airways at the time of re-exposure to Mtb antigens [3, 4]. Gene expression studies involving depletions of T cells subsets has also indicated that the local CD4 T cell-dependent expression signature centers around the multiple functions of IFN-γ [5]. More recently we have begun to explore the utility of proteomic approaches to add to our understanding of local immunity to Mtb. Comparative proteomic analysis of whole cell lysates using unlabeled mass spectrometry provides a wide-angle snapshot of the relative abundance of thousands of proteins that can be probed for differences in known functional pathways and networks and suggest novel biomarker targets. In a previous study, we found that, despite the relatively limited numbers of cells obtainable via BAL, this shotgun proteomic approach could be effectively applied to these samples to reveal distinct proteomes of human AM and MN [6]. Of unique proteins identified, 63% were shared by AM and MN, whereas 26% were identified only within MN and 11% were unique to AM. In the current study we sought to model T-cell activation of human AM and MN in LTBI using physiological levels of IFN-γ to compare proteomic responses in cells from healthy Mtb- naïve subjects. As compared to matched MN samples, our findings demonstrated surprisingly limited responses of AM to IFN-γ induced activation, despite the fact that low-dose IFN-γ stimulated early activation events of these pathways. This finding could not be accounted for by differences in one potential downstream inhibitor, suppressor of cytokine signaling 1 (SOCS1) [7].

## Materials and methods

### Subject population

Healthy volunteers (non-smokers between 18 and 50 years old) donated blood for monocyte isolation and underwent bronchoscopy with bronchoalveolar lavage (BAL) to obtain AM. Ten donors contributed samples for the exploratory proteomic analysis and 4 additional donors were recruited for validation studies. Subjects were confirmed as Mtb-naïve based on PPD skin testing or IGRA blood tests. Bronchoscopies were performed in the Dahms Clinical Research Unit of University Hospitals Cleveland Medical Center using previously described protocols [3]. All subjects consented to protocols approved by the Institutional Review Board of Case Western Reserve University and the Louis Stokes Cleveland Department of Veterans’ Affairs Medical Center.

### AM and MN isolation

BAL fluid was aliquoted into 50 mL polypropylene tubes and centrifuged at 300 x g for 10 min. Supernatants were removed, and BAL cells were resuspended in Iscove’s Modified Dulbecco’s Medium (IMDM) with 1% penicillin G. BAL cell differentials were determined by light microscopy counting of 300 cells on Wright-Giemsa–stained cytospin preparations (LeukoStat; Fisher Diagnostics, Pittsburgh, PA). The percentage of AM cells among BAL cells was > 90% by light microscopy counting of 300 cells on Wright-Giemsa–stained cytospin preparations (LeukoStat; Fisher Diagnostics, Pittsburgh, PA) and no further purification was done. Given the 90%+ purity, will refer to BAL cells as AM.

Peripheral blood mononuclear cells (PBMC) were isolated from whole blood by Ficoll-Hypaque centrifugation (GE Healthcare) and processed according to manufacturer’s instructions. For proteomic analysis, monocytes were isolated by adherence to polystyrene plates, followed by additional removal of non-adherent lymphocytes via light vortexing of the plates. For validation samples, CD14+CD16- monocytes were isolated from peripheral blood mononuclear cells using immunomagnetic negative selection (EasySep Human Monocyte Isolation Kit, Stem Cell Technologies).

### IFN-γ stimulation

AM and MN cells (1–2 x 10^6^) were re-suspended in IMDM with 2% fetal bovine serum and 1% penicillin G, aliquoted into 1.5 ml microfuge tubes, and incubated at 37°C in media with or without 0.5 ng/ml (1.0 unit/ml) human IFN-γ (ThermoFisher Scientific) at time points ranging from 5 minutes to 24 hours. This dose, while considerably lower than that used in many studies of macrophage activation by IFN-γ, was chosen as being physiologically relevant based on assessment of its production in human subjects with latent tuberculosis infection (LTBI) in response to re-exposure to Mtb antigens of purified protein derivative (PPD, 10μg/mL); further, this dose induced production of IFNγ-inducible chemokine CXCL9 in BAL samples from both LTBI and Mtb naïve individuals.

For qPCR validation experiments, AM and MN cells were cultured overnight at 37°C in 1.5 ml microfuge tubes at 8.5x10^5^/mL in complete IMDM media or with 0.5, 5, or 50 ng/mL human IFN-γ. Post incubation, cells were spun, supernatants saved, and cell pellets frozen at -80°C until RNA isolation.

### Proteomic analysis sample preparation

Samples were centrifuged at 300 x g for 10 min. Supernatants were harvested for CXCL9 chemokine measurements. Cell pellets for mass spectrometry measures were snap frozen by immersion in liquid nitrogen for 5 min prior to storage at -80°C. When sample collection was complete, cell pellets were thawed and processed simultaneously. 100 μL of 4% SDS with 1X protease inhibitor cocktail (Sigma-Aldrich, St. Louis, MO) was added to cells. Each sample underwent three 5-second pulse sonications with a probe sonicator at 15% amplitude and 5 second rest intervals between repetitions. Lysates were then incubated on ice for 45 min and pulse-sonicated again before an additional 5.5 h incubation on ice.

Following cell lysis, samples were processed using a filter-aided sample preparation (FASP) cleanup protocol with Amicon Ultra MWCO 3K filters (Millipore, Billerica, MA) as previously described (Neufeld 1991 Ann Rev Biochem). Samples were reduced and alkylated on the filters with 10mM dithiothreitol (Acros, Fair Lawn, NJ) and 25mM iodoacetamide (Acros, Fair Lawn, NJ), respectively; after which they were concentrated to a final volume of 40μL in 8M Urea. Protein concentration was performed using the Bradford Method as adapted to the manufacturer’s instructions (Bio-Rad, Hercules, CA).

Five micrograms of total protein were aliquoted for enzymatic digestion. Urea concentration was adjusted to 4M using 50mM Tris pH 8 and proteins were digested with mass spectrometry grade lysyl endopeptidase (Wako Chemicals, Richmond, VA) in an enzyme/substrate ratio of 1:20 for 2 hours at 37°C. Urea concentration was further adjusted to 2M using 50mM Tris pH 8 and Lysyl peptides were additionally digested with sequencing grade trypsin (Promega, Madison, WI) in an enzyme/substrate ratio of 1:20 at 37°C overnight. Finally, samples were diluted in 0.1% formic acid (Thermo Scientific, Rockford, IL) prior to LC-MS/MS analysis. 400fmol of Pierce^®^ Retention Time Calibration Mixture (Thermo Scientific, Rockford, IL) was spiked into samples to track retention times and mass drift across all samples. MN samples showed a maximum retention time drift of 2.07 min whereas AM samples showed a 3.49 min maximum retention time drift across the same 9 tracked peptides. Additionally, the mass drift was accounted for using the same 9 tracked peptides in both cell types; MN showed a maximum 4.15 ppm error whereas AM had a 3.35 ppm maximum mass error.

### Liquid chromatography and mass spectrometry

For each sample, 400ng of AM and MN peptide digests were loaded on a column in a 14μL injection. Samples were randomized and blanks were added between samples. Resulting data was acquired on an Orbitrap Velos Elite mass spectrometer (Thermo Electron, San Jose, CA) equipped with a Waters nanoAcquity LC system (Waters, Taunton, MA). Peptides were desalted in a trap column (180 μm × 20 mm, packed with C18 Symmetry, 5μm, 100Å, Waters, Taunton, MA) and subsequently resolved in a reversed phase column (75μm x 250 mm nano column, packed with C18 BEH130, 1.7μm, 130Å (Waters, Taunton, MA)). Liquid chromatography was carried out at ambient temperature at a flow rate of 300 nL/min using a gradient mixture of 0.1% formic acid in water (solvent A) and 0.1% formic acid in acetonitrile (solvent B). The gradient employed ranged from 4 to 44% solvent B over 210 min. Peptides eluting from the capillary tip were introduced into the nanospray mode with a capillary voltage of 2.4 kV. A full scan was obtained for eluted peptides in the range of 380–1800 atomic mass units followed by twenty-five data dependent MS/MS scans. MS/MS spectra were generated by collision-induced dissociation of the peptide ions at normalized collision energy of 35% to generate a series of b- and y-ions as major fragments. A one-hour wash was included between each sample.

### Protein identification

Progenesis LC-MS version 4.1 software (Nonlinear Dynamics, Garth Heads, UK) was used to align the retention times (using Progenesis-recommended cut-offs), normalize and identify data features. The IPI-human database from June 2010 (86,392 sequences) was used as a decoy database. FDR was determined by assessment of peptide matches above the identity or homology threshold. Key search parameters were Trypsin + Lys-C for enzyme; a maximum of 2 missed cleavages; peptide charge states of +2 to +4; peptide tolerance of 10 ppm; and MS/MS tolerance of 0.8 Da. Fixed modifications included carbamidomethylation of cysteine residues. Oxidation of methionine was a variable modification.

Lists of all measured peptides including sequences, charge states, modifications and protein identifiers are available in the supporting information.

### ELISA

Culture supernatants were collected and stored after IFN-γ stimulation. CXCL9 chemokine concentration was measured using a commercially available ELISA kit (R and D Systems) according to manufacturer’s instructions.

### Western blot analysis

1 x 10^6^ MN and AM cells from each donor were cultured with recombinant human IFN-γ (0.5 ng/ml) overnight. Cells were pelleted and lysed in RIPA buffer (Cell Signaling Technologies), supplemented with Halt Protease and Phosphatase Inhibitor Cocktail (ThermoFisher Scientific), sonicated for 1 minute and centrifuged at 14,000 RPM for 10 minutes at 4°C. Protein concentration in lysates was measured using the Pierce BCA Protein Assay kit (ThermoFisher Scientific) and diluted to a concentration of 1–2 μg/uL. Samples were heated to 95°C for 5 minutes in Laemmli buffer (Alfa Aesar), separated on a Novex 4–20% Tris-glycine gel (ThermoFisher Scientific) and transferred to an Immuno-Blot PVDF membrane (BioRad Laboratories). Membranes were blocked in 5% BSA with 1% Tween 20 for 1 hour then probed overnight at 4°C with the following primary antibodies at a 1:1,000 dilution: Phospho-STAT1 (Tyr 701) (Cell Signaling Technologies, 9167S, monoclonal), SOCS1 (Cell Signaling Technologies, 3950S, polyclonal), STAT-1 (Cell Signaling Technologies, 14994S, monoclonal), IFNGR1 (Abcam, 134070, monoclonal) and GAPDH (Cell Signaling Technologies, 2118S, monoclonal). The next day, membranes were washed 3 times in TBST (Tris buffered saline with 0.1% Tween20) for 10 minutes and probed for 1 hour at room temperature with the horseradish peroxidase-conjugated mouse anti-rabbit IgG monoclonal antibody at a 1:5,000 dilution (Jackson ImmunoResearch, 211-035-109). Membranes were washed three more times in TBST before protein expression was detected using the Pierce ECL Plus Substrate (ThermoFisher Scientific) on the BioBlot Blue X-Ray film (Laboratory Product Sales). Membranes were cut to probe a single blot with 2 antibodies simultaneously and/or stripped and reprobed with additional target protein antibodies. Protein/antibody intensity bands for each target were measured relative to GAPDH from the same blot. Bands were quantified using ImageJ software (version 1.53a; available from website imagej.nih.gov) and normalized to the GAPDH loading control. All raw western blot images are available in the S1 Raw images.

### mRNA measurement by quantitative PCR

Total RNA was purified from the MN and AM (0.5 x 10^6^ cells) using the RNeasy Mini Kit (Qiagen). Total RNA was reverse transcribed using the QuantiTech Reverse Transcription Kit (Qiagen). RT-qPCR samples were run in triplicate on the StepOne Plus Real-Time PCR System (Applied Biosystems) at 95°C for 20 minutes, then 40 cycles of 95°C for 1 minute, concluding with a 60°C cycle for 20 minutes. The total reaction volume was 20 ul per well. Each well contained 17 μl of TaqMan Fast Advanced Master Mix (Applied Biosystems), 0.75 μl of the forward and reverse primer (OriGene, HP205798), and 1 μl primer pair (Thermo Fisher) and 2 μl of cDNA. Primer pairs are described in Table 1.

**Table 1:** Primers for reverse transcription quantitative PCR.

mRNA target	Thermo Fisher Primer Pair
*GAPDH*	Hs02786624_g1
*STAT-1*	Hs01013996_m1
*CXCL9*	Hs00171065_m1
*TAP2*	Hs00241060_m1

### Data analysis

**Fig 1:**
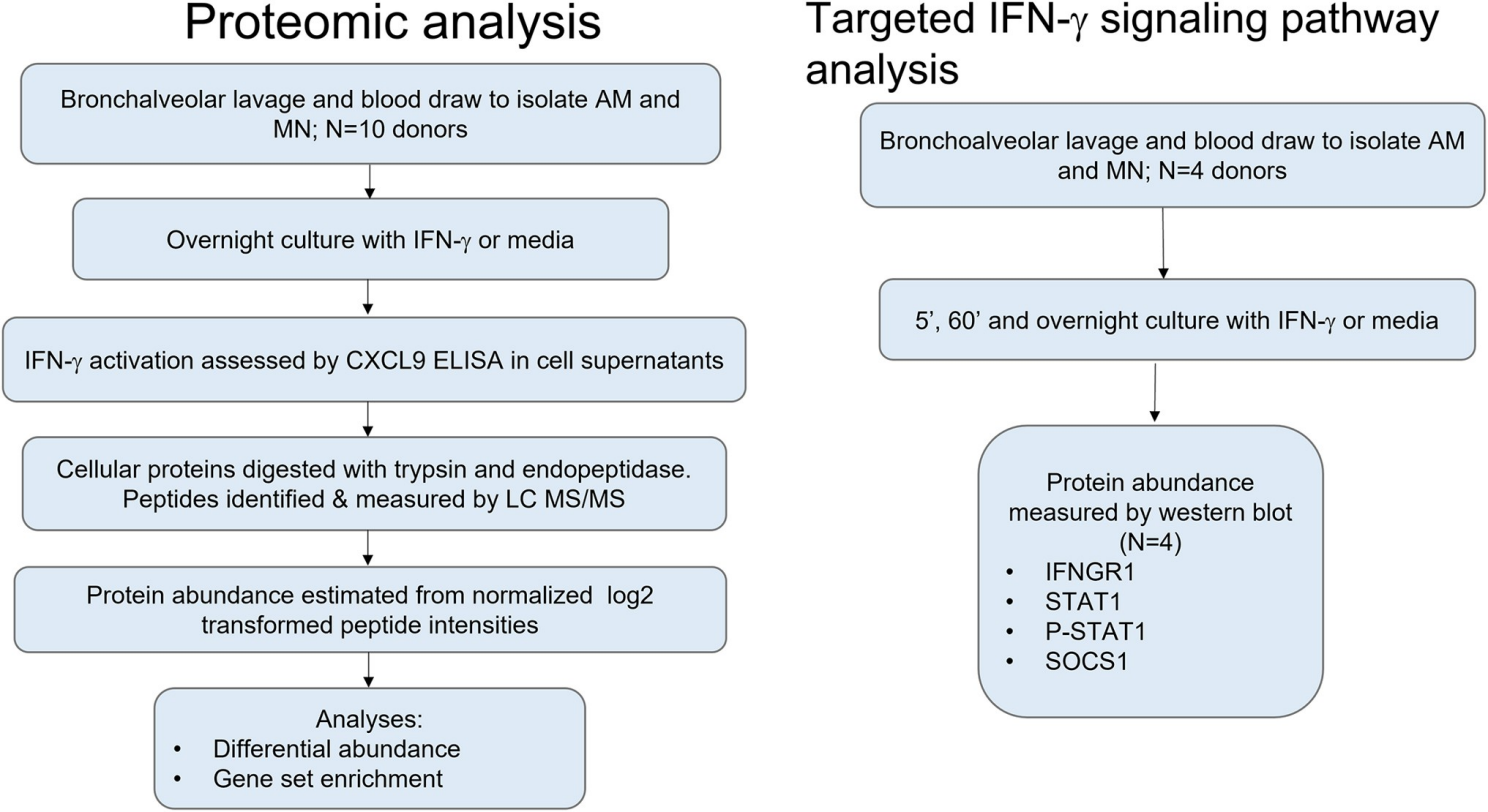
Workflow. Workflow for exploratory proteomic analysis of autologous AM and MN samples stimulated with media or IFN-γ and the independent validation of STAT1 signaling and SOCS1 protein expression in AM and MN.

Data integration and analysis pipeline is shown in Fig 1. Data summarization and statistical analyses were done using R (Versions 3.2.3 and 3.5.1, Vienna, Austria). For the proteomic dataset, proteins identified by a single peptide in only one cell type were filtered out of the analysis dataset as these have a high probability of being miss-identified proteins. A linear modeling approach was used to estimate the effect of IFN-γ on protein levels while controlling for the correlated peptide abundance measures within each subject [8, 9]. Treatment effect t-statistics, estimated for MN and AM separately, were ranked by the size of the IFN-γ effect and tested for significant enrichment of functionally related proteins using gene sets from the Molecular Signatures Database (MsigDB, version 7.2 accessed November 2020) where the term ‘gene’ refers to a gene-product [10]. The Hallmark set of molecular signatures was used to test for enrichment in the IFN-γ response process [11].

Protein abundance was quantified by western blot band intensity relative to GAPDH band intensity. The relative intensity was assumed to be normally distributed and varying subject baselines were accounted for using the paired t-test comparing unstimulated and stimulated samples. Transcript mRNA abundance was measured by qPCR was normalized to GAPDH levels using the -2ΔCt method, and a paired t-test was used to test for a significant IFN-γ effect. Analyses were done in R (Version 4.0.4, Vienna, Austria). Results were graphed in Graphpad Prism for Windows V9.1.0 (San Diego, CA).

## Results

### Overnight stimulation of AM and MN with 0.5 ng/ml IFN-γ is sufficient to induce an increase in *STAT1* and *CXCL9* mRNA and MIG (CXCL9) protein

**Fig 2:**
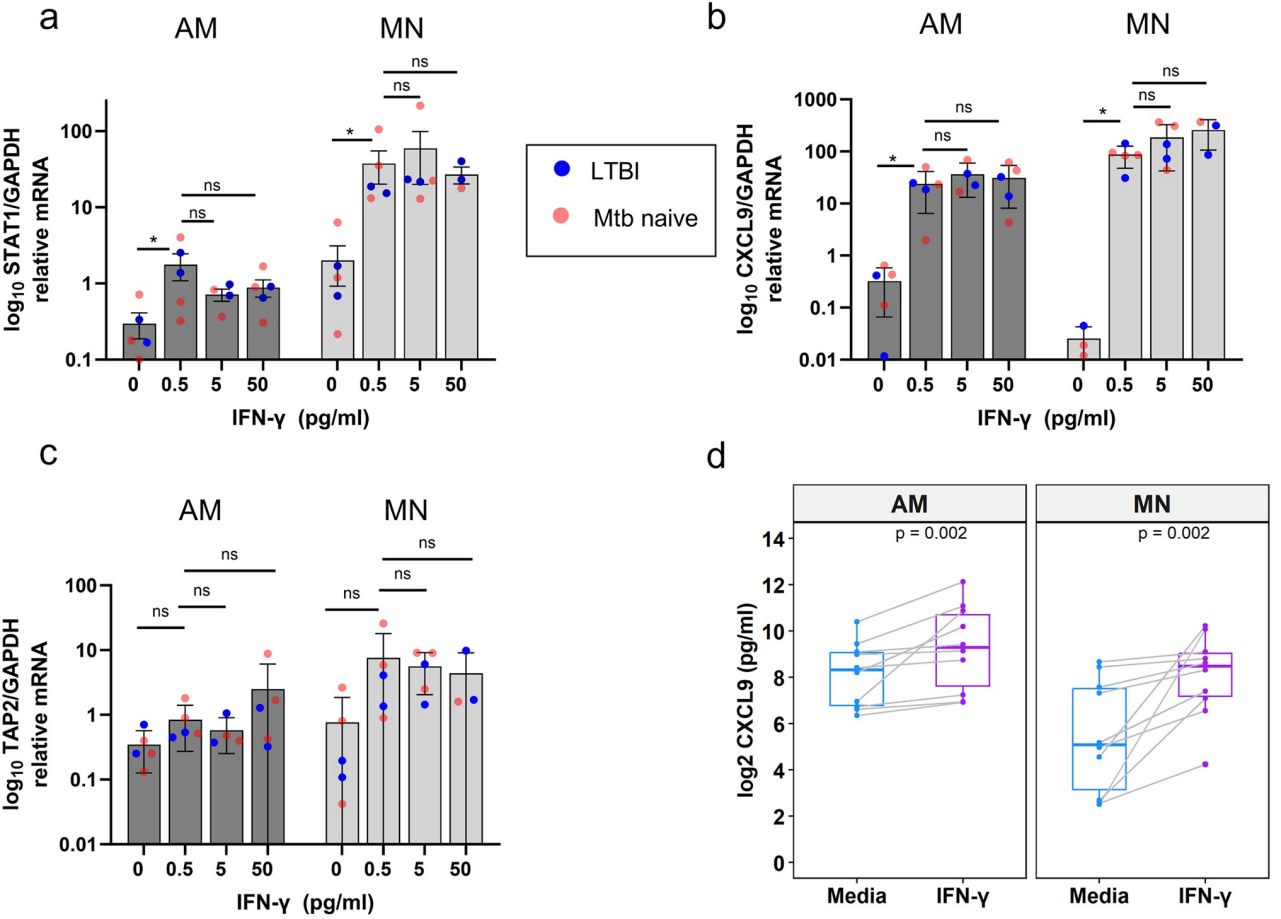
Assessment of IFN-γ stimulated transcription of canonical interferon stimulated genes and chemokine secretion indicates that AM responses are preserved across a wide range of doses and do not differ between LTBI and Mtb-naïve subjects. Expression of IFN-γ inducible genes (as ratio of mRNA relative to *GAPDH*) following stimulation with concentrations of 0.5, 5.0, and 50 ng/mL are illustrated for LTBI and Mtb-naïve subjects (as red and blue circles, respectively). There was no indication that the infection status of the donors was related to the IFN-γ response. a) *STAT1* transcript was significantly increased in AM and MN at the 0.5 ng/ml dose. Transcript levels in MN were more than an order of magnitude greater than AM and therefore the y-axis is shown on a log scale. b) *CXCL9* transcript was significantly increased in AM and MN at the lowest dose and did not increase with increasing dose. d) *TAP2* mRNA increase at 0.5 ng/ml did not reach statistical significance. d) CXCL9 (MIG) chemokine levels were measured in AM and MN culture supernatants by ELISA with and without overnight IFN-γ stimulation. Lines are shown connecting the paired results for each donor.

For experiments evaluating the impact of IFN-γ on the AM and MN proteome, we utilized an IFN-γ concentration (1 U/ml;0.5 ng/ml) based on levels measured in the supernatants of similar cultures of BAL cells from individuals with latent tuberculosis infection (LTBI) following overnight incubation with PPD (10μg/mL) [3]. Many studies of IFN-γ induced pathways have used substantially higher doses. In addition, macrophage conditioning in LTBI donors could lead to a lower threshold for IFN-γ induced stimulation than seen in AM from control subjects. We therefore assessed the effect of increasing IFN-γ dose on transcription of canonical IFN-γ genes using paired MN and AM from 3 Mtb-naïve and 2 LTBI donors with quantitative PCR. Fig 2 shows the relative gene expression after overnight treatment with 0, 0.5, 5 and 50 ng/ml IFN-γ. There was significant upregulation of *STAT1* and *CXCL9* transcripts in both AM and MN, and no indication of a difference between LTBI and Mtb naïve donors. The largest effect occurred in the 0.5 ng/ml dose with no significant increases in transcript with increasing dose. A third gene known to be activated by IFN-γ, *TAP2*, showed a similar pattern but did not reach significance. IFN-γ-induced activation was also confirmed by measuring IFN-γ -dependent chemokine CXCL9 (MIG) by ELISA in overnight culture supernatants. Paired AM and MN from 10 healthy Mtb-naïve donors (the same donor samples used for proteomic studies) were measured in response to IFN-γ (Fig 2d; p = 0.002) indicating that the AM cells were responsive to physiologic doses of IFN-γ. Overall, these experiments demonstrate that a low dose of IFN-γ induces a significant but smaller activation in AM relative to MN cells, re-enforcing the validity of the physiologic dose we chose for the proteomic assessment.

### The global AM response to IFN-γ is markedly less robust than that of MN

**Fig 3:**
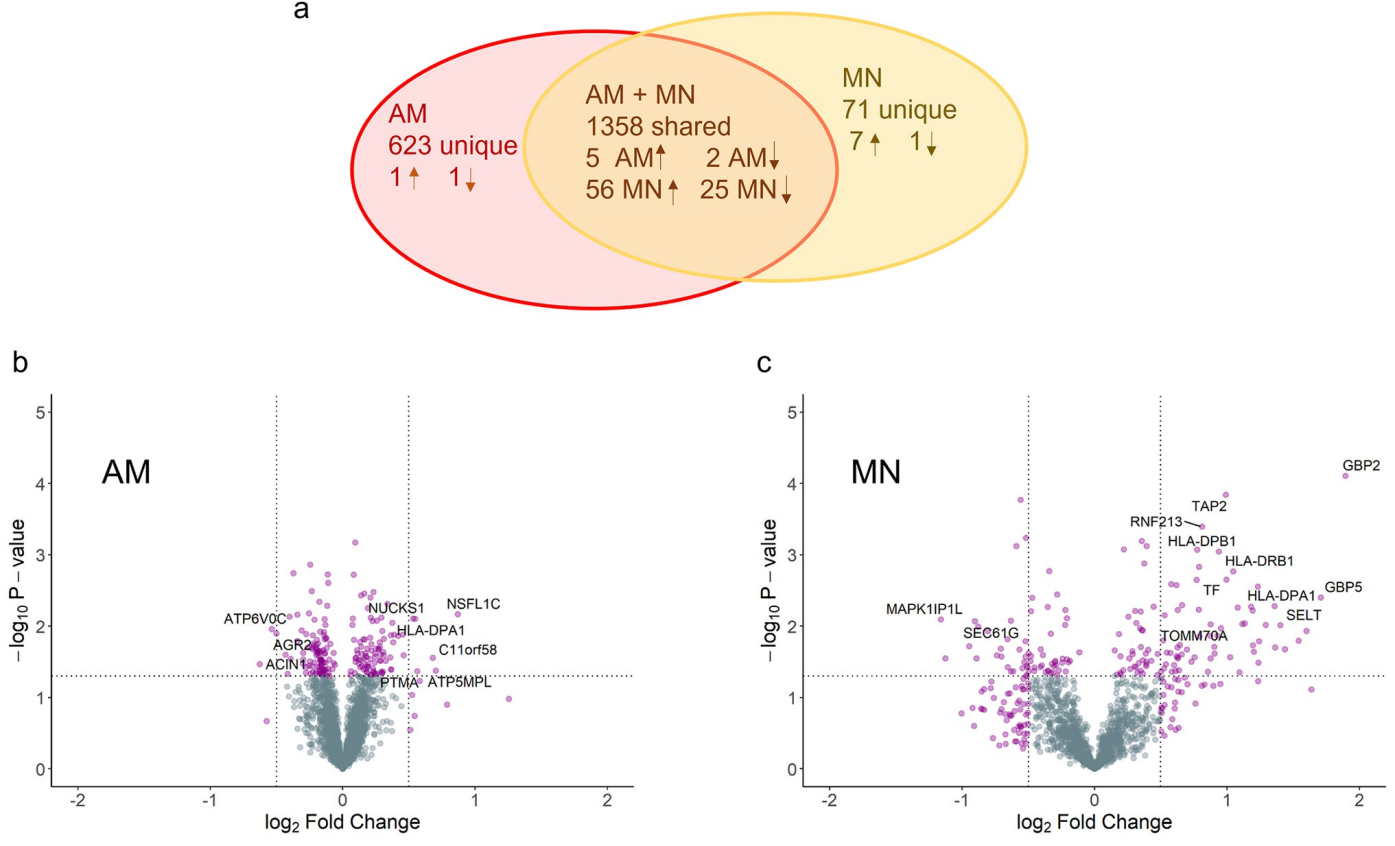
IFN-γ stimulation of AM and MN cells for 16–24 hours results in a larger number and percentage of differentially abundant proteins in MN relative to AM. a) Venn diagram with the number of proteins discovered within and across both cell types and the number of proteins up- and down-regulated based on *p*-value < 0.05 and absolute value fold change (FC) > 0.5. b) MN and c) AM volcano plots showing the significance (-log_10_ *p*-value) vs the log_2_ fold change in protein abundance. Data reflect the results from paired MN and AM from 10 healthy adult volunteers.

The 10 matched donor AM and MN samples with and without IFN-γ stimulation were measured by LC MS/MS. Runs for all AM samples were aligned and aggregated such that there were no missing values, yielding 16,346 peptides mapped to 2,538 unique proteins under both stimulated and unstimulated conditions. Similarly, for the MN samples, 7,199 peptides were identified and mapped to 1,553 proteins. The median number of peptides discovered per protein in the combined AM and MN datasets was 6 (min = 1, max = 184). After filtering out proteins identified by only in a single peptide across both AM and MN datasets, there were 1981 AM and 1429 MN proteins. Fig 3a shows the number of proteins found within and across AM and MN including the number of significantly differentially abundant proteins.

Testing each protein for a significant IFN-γ treatment effect yielded 89 MN proteins and 9 AM proteins using a *p*-value < 0.05 and absolute fold change (|FC|) > 0.5 as significance criteria. A list of all filtered proteins with estimated treatment effects and *p*-values are available in the supporting information. Of the 9 differentially abundant AM proteins, 6 increased in and 3 decreased in abundance, in contrast, in MN 63 proteins increased and 26 decreased in response to IFN-γ. Volcano plots for AM and MN proteins are shown in Fig 3b and 3c. The 6 significantly increased proteins in AM were ATP5MPL, C11orf58, HLA-DPA1, NSFl1C, NUCKS1 and PTMA. The 3 significantly downregulated proteins were ACIN1, AGR2, and ATP6VOC. For MN the top proteins with increased abundance included GBP2, TAP2, HLA-DPB1, HLA-DPA1, HLA-DRB1 and STAT1. The most downregulated MN proteins included MAPK1IP1L, SEC61G and CALM5.

### Differential identification of IFNγ-induced pathway proteins in AM and MN

**Fig 4:**
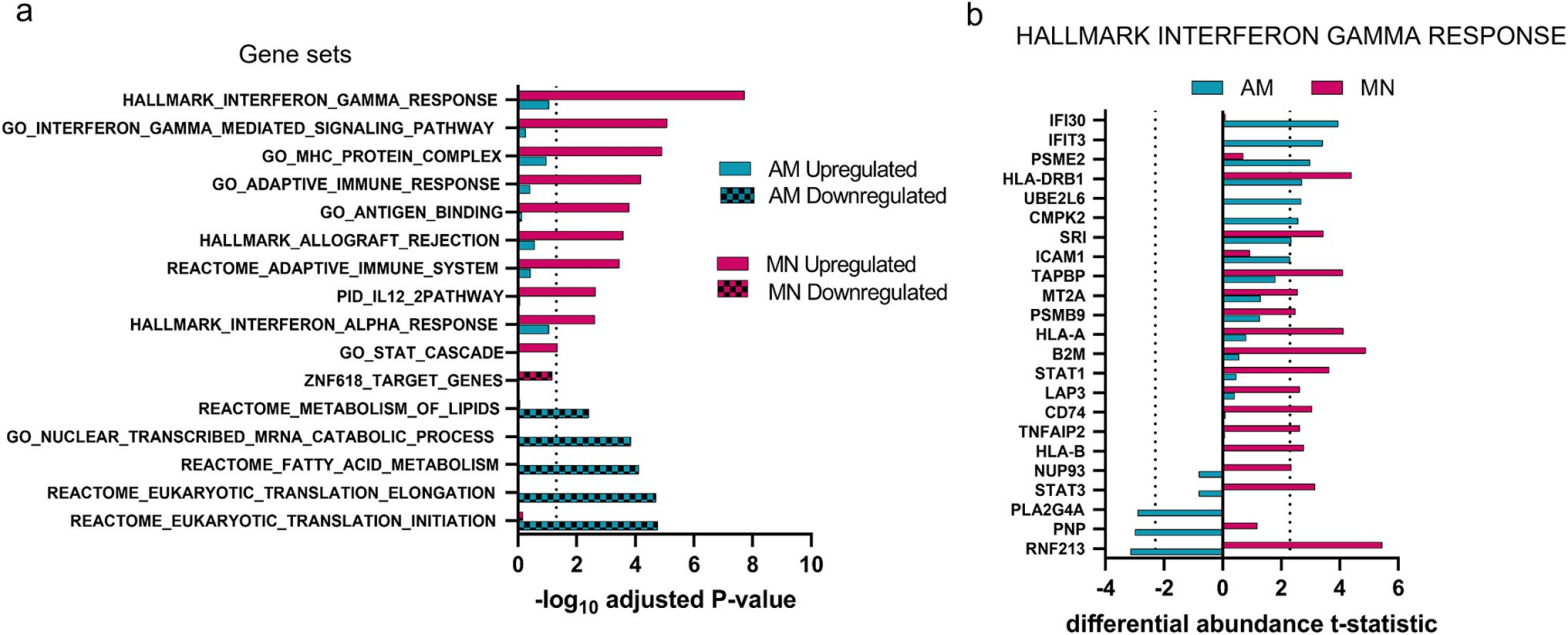
IFN-γ response signatures were significantly enriched with upregulated MN proteins in contrast to AM proteins which showed no significant upregulated enrichment. a) AM and MN proteins were ranked based on the IFN-γ differential abundance effect size and analyzed for signature enrichment. Dotted line at 1.3 delineates enrichment scores with adjusted *p*-value < 0.05. b) AM and MN proteins within the most significantly enriched gene set, *Hallmark Interferon Gamma Response*, illustrate that more proteins in this set are differentially regulated in MN (14) relative to AM (6 upregulated, 3 downregulated). The x-axis shows the differential abundance t-statistic for each protein with dotted lines at significance level = 0.05.

To identify functional relationships between proteins regulated by IFN-γ in each cell type, we tested for significant enrichment of MsigDB gene sets among all proteins ranked by the amount of differential abundance as measured by the t-statistic. Fig 4a shows a comparison of gene sets enriched among AM and MN ranked proteins. The top enrichment in MN contained upregulated proteins involved in IFN-γ and cytokine responses, whereas in AM these same gene sets were upregulated to a lesser extent and did not reach statistical significance for enrichment. The only gene sets found to be significantly enriched among AM proteins were downregulated and related to lipid modification and catabolic processes.

To determine if proteins in canonical IFN-γ pathways were absent from AM cells or simply had lower abundance changes relative to MN, we focused on the proteins within the *Hallmark IFN-*γ *Response* gene set which is a curated collection of genes involved in IFN-γ regulated pathways and processes. As shown in Fig 4b, many of the proteins that were upregulated in MN were found in AM but had smaller IFN-γ responses. In fact, several proteins in the canonical IFN-γ response were downregulated in AM. In summary, we expected IFN-γ to significantly alter the abundance of proteins involved in the classical IFN-γ regulated immune response but found that at a proteomic level, AM cells were minimally responsive. In contrast, MN were much more responsive to low levels of IFN-γ stimulation.

### More limited IFN-γ-induced proteomic responses of AM vs MN are not explained by deficient IFN-γ signaling

**Fig 5:**
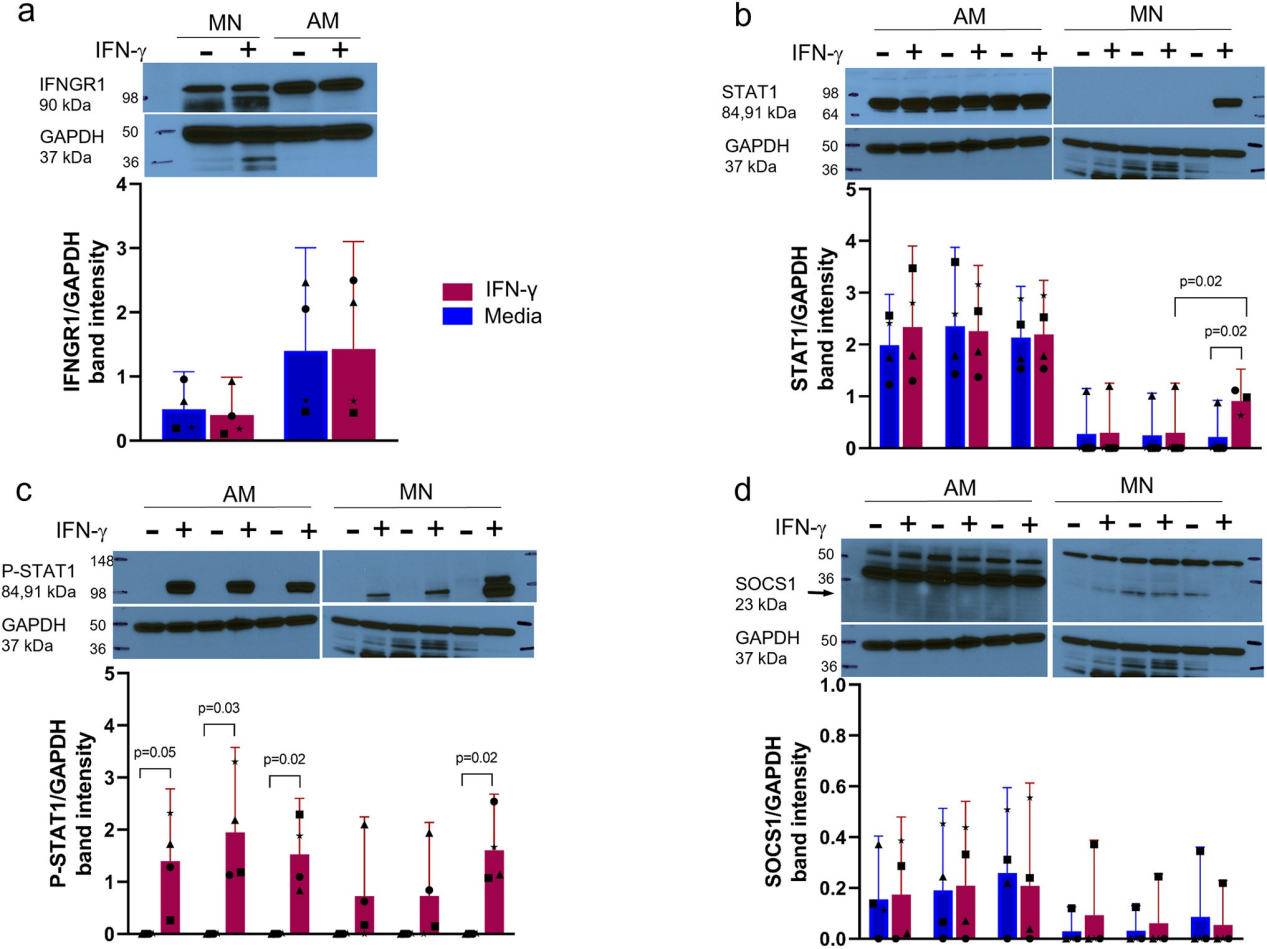
Differences in the proteomic outcomes of IFN-γ stimulation of AM vs MN are not associated with major functional proteins of the IFN-γ proximal signaling pathway. a) IFNGR1/GAPDH relative protein abundance in MN and AM with and without overnight (o/n) IFN-γ stimulation shows robust receptor levels that were not associated with treatment. b) STAT1/GAPDH relative protein abundance over time in AM and MN in the presence and absence of IFN-γ show abundant constitutive STAT1 levels in AM and up-regulation of STAT1 in MN following overnight treatment. c) P-STAT1/GAPDH protein abundance in AM and MN show phosphorylation of STAT1 (Tyr701) in response to IFN-γ and upregulation of in MN after overnight treatment. d) SOCS1/GAPDH protein abundance time course in the presence and absence of IFN-γ showed low levels that were not associated with treatment. Of note, due to limited cell numbers obtainable from human BAL procedures, Fig 5b-5d were performed with a single blot each for AM and MN studies, accounting for the identical GAPDH bands throughout. Blots were stripped before re-use with alternate probes. Band intensities were normalized to GAPDH levels (N = 4). Representative western blots are shown above the plots. Bar plots are mean +/- 95% CI. Symbol shapes (●■▲_*_) represent individual donors.

The diminished response in AM to IFN-γ relative to MN suggested the possibility that the proximal IFN-γ signaling pathway was inhibited or less activated in AM. We measured IFNGR1, P-STAT1, STAT1 and SOCS1 by western blot to look for differences in AM and MN protein levels over time. IFNGR1 levels were similar in AM and MN before and after IFN-γ stimulation (Fig 5a). In AM, STAT1 levels were constitutively higher relative to MN and not increased by IFN-γ stimulation (Fig 5b). In contrast, constitutive STAT1 protein abundance was substantially lower in MN, but increased significantly overnight (p = 0.02). However, examination of phosphorylated STAT1 (P-STAT1) (Fig 5c) demonstrated IFN-γ induced activation at all time points in AM, but was only increased significantly in MN overnight (p = 0.02). Levels of the SOCS1 repressor protein were low in both cell types and there were no constitutive differences or significant changes with IFN-γ stimulation (Fig 5d).

The hypo-responsiveness in AM relative to MN is not due to a decrease in proximal signal transduction via JAK-STAT1 as assessed via both STAT1 transcription and STAT1 phosphorylation; nor is it due to an upregulation of the SOCS1 cytokine suppressor protein.

## Discussion

In this study, we utilized a proteomic approach to assess the global impact of IFN-γ stimulation of primary human AM and MN as a means to model local Th1 adaptive immune responses in LTBI. Accordingly, this was based on a physiologic dose of IFN-γ (0.5 ng/ml, or 1.0 unit/ml) as we had observed in previous studies involving stimulation of BAL cells from LTBI subjects with Mtb protein antigens in the form of PPD. Total proteomic abundance changes were measured using unlabeled mass spectrometry to provide a comprehensive view of the protein interactions and pathways in an overnight IFN-γ activation snapshot. At the proteomic level, a similar number of proteins were detected in both cell types; however, IFN-γ treatment had a significant effect on and the abundance of 89/1429 in MN but only 9/1981 proteins in AM. Gene set enrichment analysis showed that proteins involved in the classic IFN-γ immune response were significantly enriched among the upregulated MN proteins showed little enrichment in AM. This effect may be due to an active down-regulated response in AM cells or the absence of the ability to mount a sustained inflammatory response. Further investigation explored potential mechanisms for these differences at multiple levels, based on several elements of the IFN-γ response pathway, including phosphorylation of STAT1 protein following engagement of the dimeric IFN-γ receptor, induction of downstream STAT1 protein, and possible downregulation by the SOCS1 repressor protein.

The combined approaches of shotgun proteomics, Western blot, and mRNA expression indicated complex differences in the initial responses of AM and MN to IFN-γ stimulation. We found a relative resistance to Th1 cytokine stimulation in human AM despite increased CXCL9 secretion in both cell types. Since AM secrete CXCL9 in response to IFN-γ stimulation and CXCL9 is known to be expressed through JAK-STAT signal transduction [12], we focused on proteins involved in STAT1 signaling to test for differential function in AM and MN using an independent set of donors. AM displayed substantially less STAT1 message than MN both at baseline and in response to a wide dose-response range of IFN-γ concentrations (0.5–50 ng/mL); nevertheless, constitutive STAT1 protein abundance was greater in AM than MN. STAT1 was also rapidly phosphorylated in AM. In contrast, MN displayed greater STAT1 message in response to IFN-γ, had lower constitutive abundance of the total protein and more delayed phosphorylation following stimulation. We also considered the possibility that SOCS1 was present in greater abundance in AM than MN as a potential mechanism. Although SOCS1 was present in greater abundance in AM than MN, it did not display a significant change in in response to IFN-γ to suggest that a more brisk activation of this negative feedback loop represented the mechanism of decreased IFN-γ responsiveness in AM. Overall, the combination of findings suggests that the limited proteomic responses to IFN-γ in AM was not due to impaired initial signaling.

Several gene sets showed significant enrichment of downregulated proteins in AM and no enrichment in MN. Downregulation of AM proteins in gene sets involved in lipid and fatty acid metabolism suggest a switch to glycolysis from oxidative phosphorylation to supply cellular energy, however this switch is generally associated with an increased inflammatory/M1 phenotype in macrophages that have been stimulated by LPS [13, 14]. It is not clear whether a decrease in proteins related to the oxidative phosphorylation pathway is associated with a global reduction in the IFN-γ stimulated response and this represents a potential area of future investigation.

Significant upregulation of a large number of proteins in AM cells was not necessarily expected given other studies of lung AM showing a tendency towards homeostasis following antigen stimulation. Previous studies have shown a suppressed response in murine AM where downregulation of bacterial clearance occurs with IFN-γ exposure [15, 16]. Less is known about the human AM response to cytokine stimulation although several studies have shown evidence that AM macrophages are poor activators of T-lymphocytes relative to autologous MN [17] possibly due to lower induction of surface co-stimulatory molecules [18]. Lung macrophages in healthy individuals do not appear to be polarized towards either an inflammatory or anti-inflammatory phenotype.

Although the procedure of bronchoscopy with BAL provides unique access to an immunologically relevant primary cell population, BAL samples of healthy individuals provide a relatively limited number of cells (averaging approximately 1x10^7^). The requirements of LC MS/MS therefore limits the number of study variables, such as doses of stimulus and time points following stimulation that can be assessed with a single sample. Further, although LC/MS/MS can measure thousands of peptides there is a limitation on measurement of low abundance and secreted proteins and therefore chemokines such as CXCL9, expected to be regulated by IFN-γ, could not be detected in the proteomics assay. Nevertheless, in combination with other approaches, this proteomic data indicated significant differences between the responses of AM and MN that are relevant to understanding initial interactions of resident airway immune cells with respiratory pathogens.

Our studies also indicated that IFN-γ stimulation itself does not fully recreate the immunologic events observed following stimulation of BAL cells from LTBI subjects with Mtb and/or its protein antigens. This finding is consistent with findings that protective Mtb-responsive cells of the airways display significant polyfunctionality and produce various combinations of TNF-α, IL-2 and CD153, among other immune mediators; further interactions of memory immune cells with resident AM are likely to include specific cell-surface interactions that further alter AM responses. Finally, re-stimulation of baseline BAL of immune individuals with Mtb antigens likely results in recruitment to the airways of additional macrophage populations that may broaden the local immune response by including bone-marrow derived phagocytes with greater capacity for pro-inflammatory function.

## Supporting information

S1 Dataset Summary measures for all proteins. (XLSX)

S1 File MN peptide abundance measures. (XLSX)

S2 File AM peptide abundance measures. (XLSX)

S1 Raw images Western blot images. (PDF)
