# Supplementary material for: Human alveolar macrophages display marked hypo-responsiveness to IFN-γ in both proteomic and gene expression analysis
Source: PLoS One. 2024 Feb 1;19(2):e0295312. doi: 10.1371/journal.pone.0295312 (PMC10833554; doi:10.1371/journal.pone.0295312)
Supplement: S1 Raw images — (PDF) [file pone.0295312.s004.pdf]

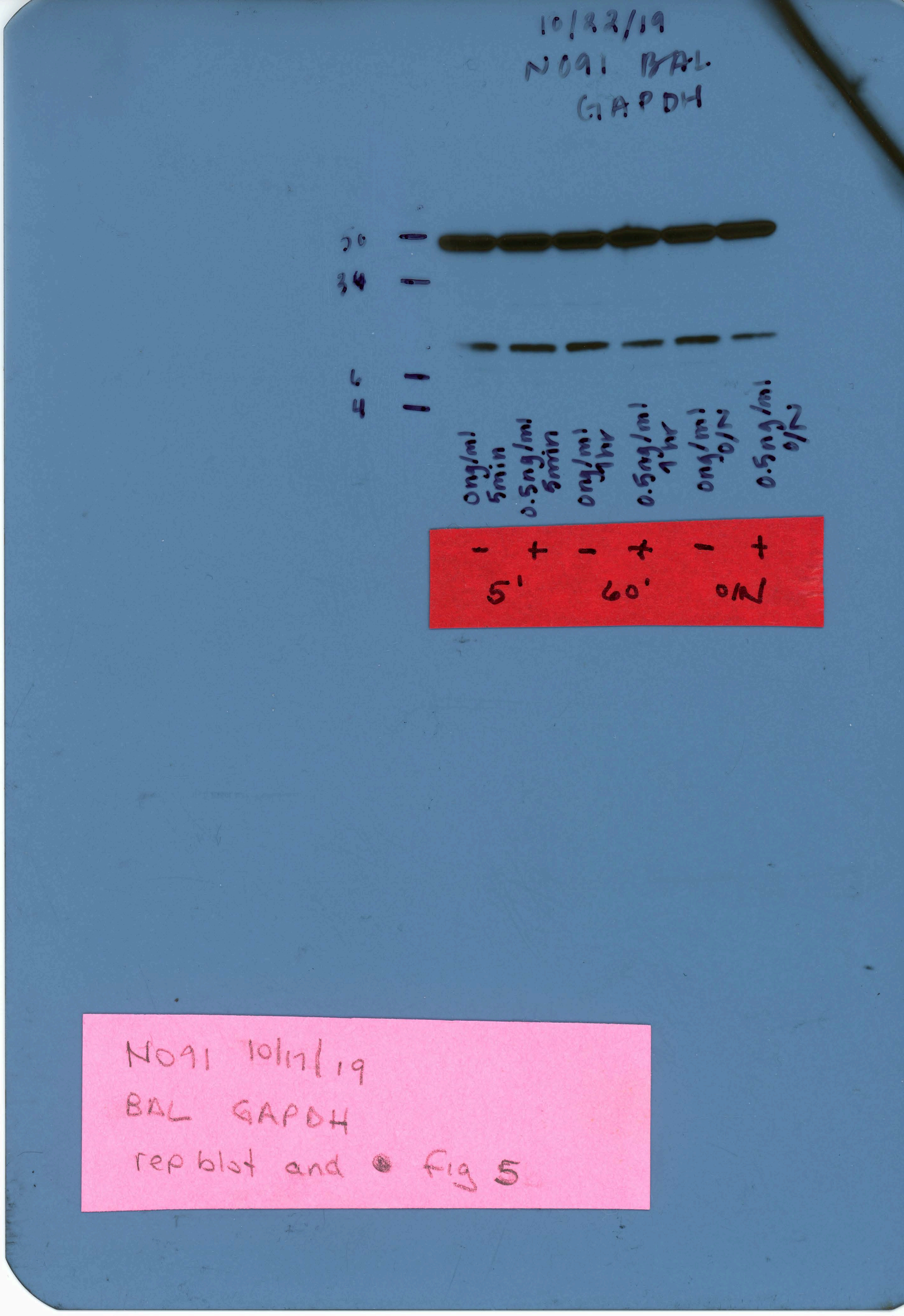
10/22/19
N091 BAL
GAPDH
50
34
6
4
0ng/ml 5min
0.5ng/ml 5min
0ng/ml 1hr
0.5ng/ml 1hr
0ng/ml O/N
0.5ng/ml O/N
- + - + - +
5' 60' O/N
N091 10/17/19
BAL GAPDH
rep blot and fig 5

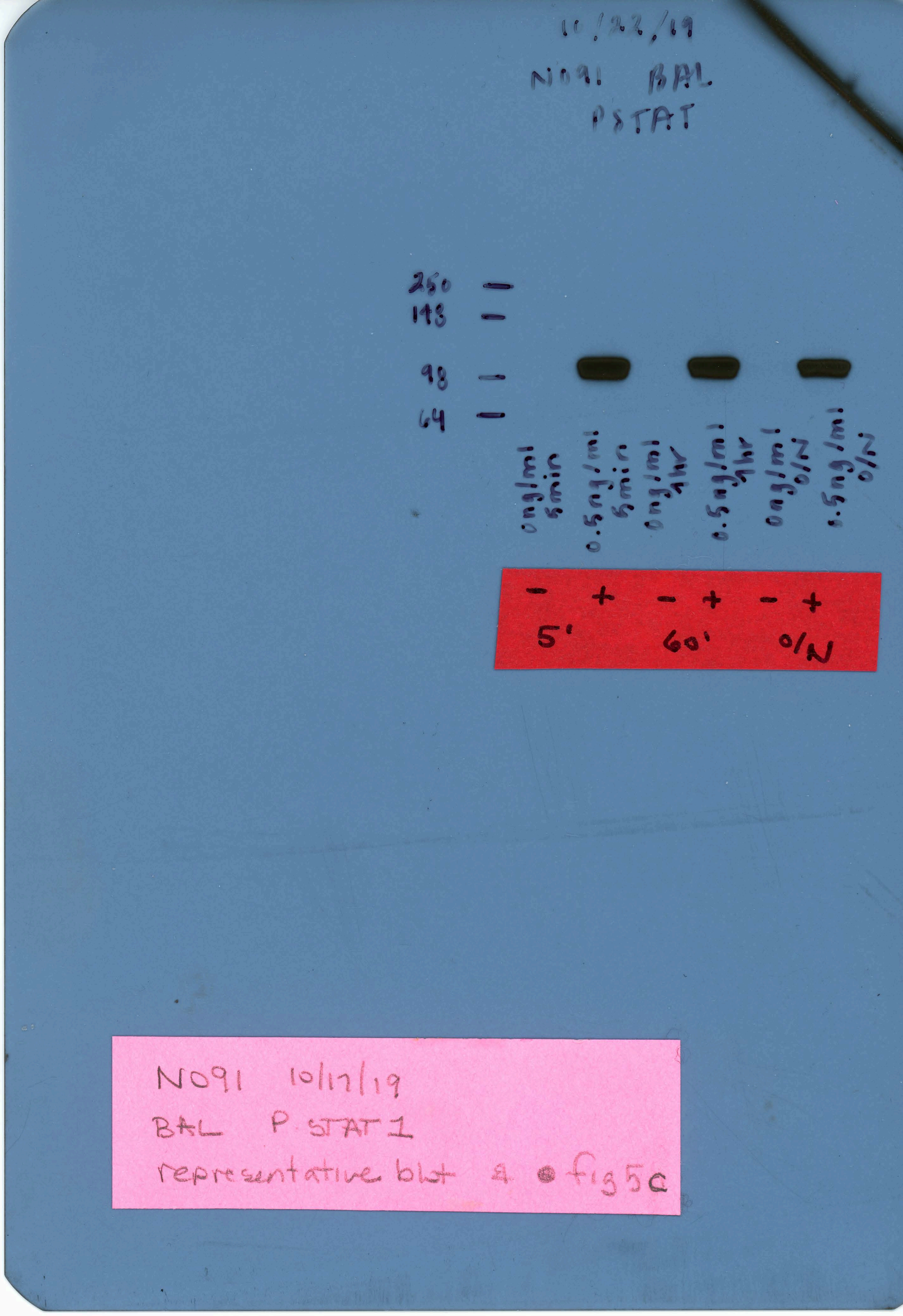
10/22/19
N091 BAL
PSTAT
250
148
98
64
0ng/ml 5min
0.5ng/ml 5min
0ng/ml 1hr
0.5ng/ml 1hr
0ng/ml O/N
0.5ng/ml O/N
- + - + - +
5' 60' O/N
N091 10/17/19
BAL P STAT1
representative blot 4 fig5c

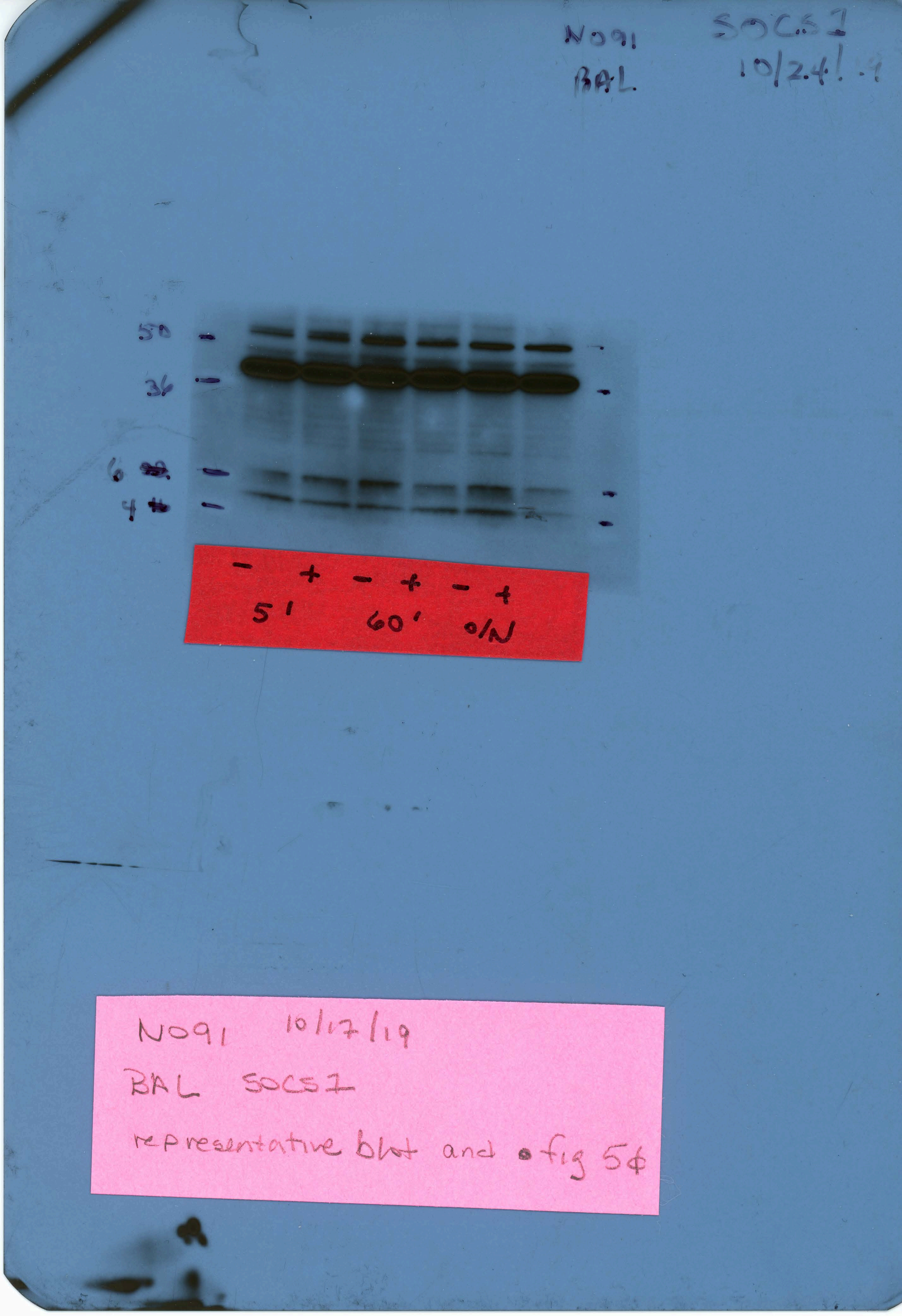

N091
BAL
SOCS1
10/24/19
50
36
6
4
- + - + - +
5' 60' o/N
N091 10/17/19
BAL SOCS1
representative blot and fig 5

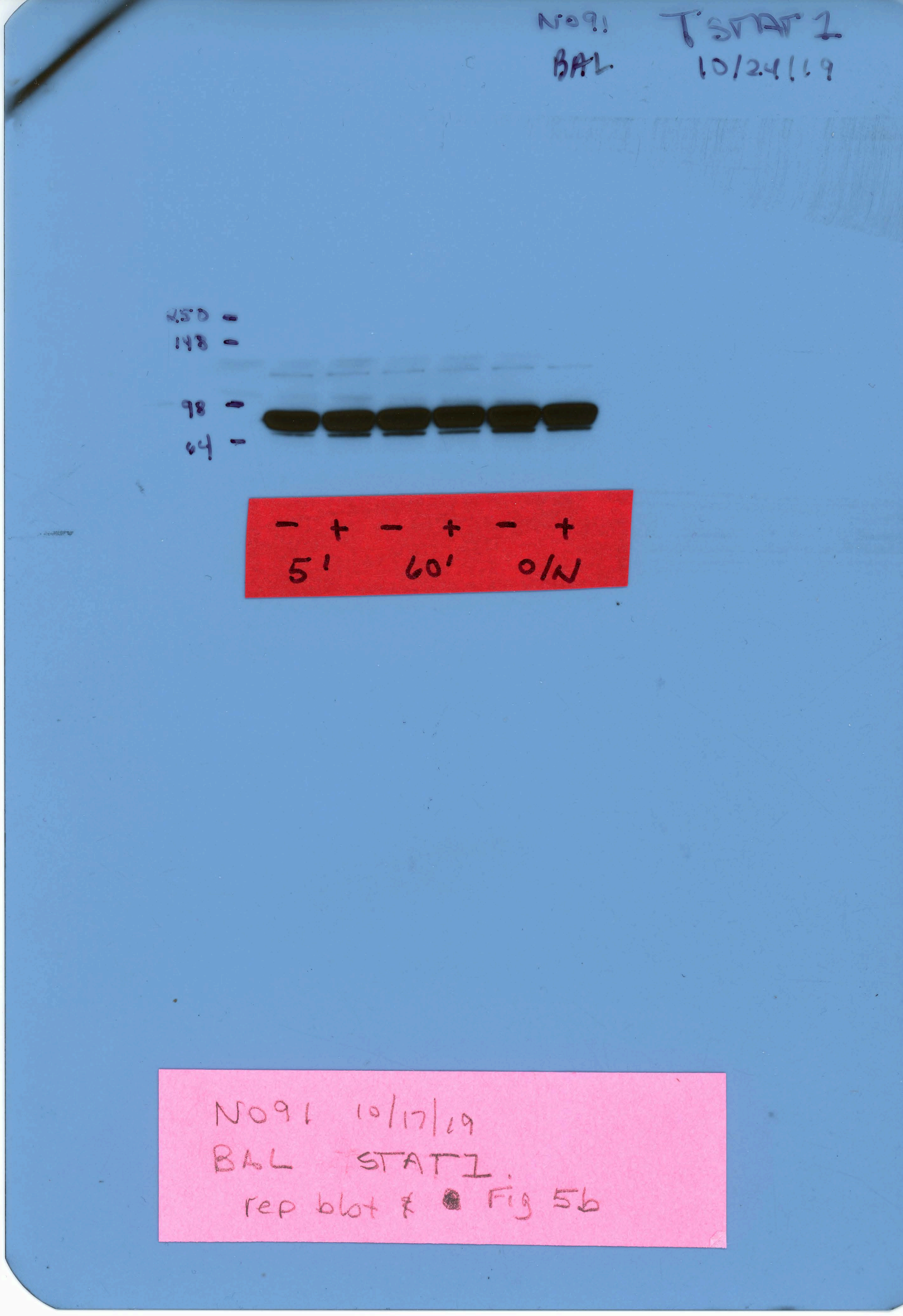

N091
BAL
T STAT 1
10/24/19
250 -
148 -
98 -
64 -
- + - + - +
5' 60' O/N
N091 10/17/19
BAL STAT1
rep blot * Fig 5b

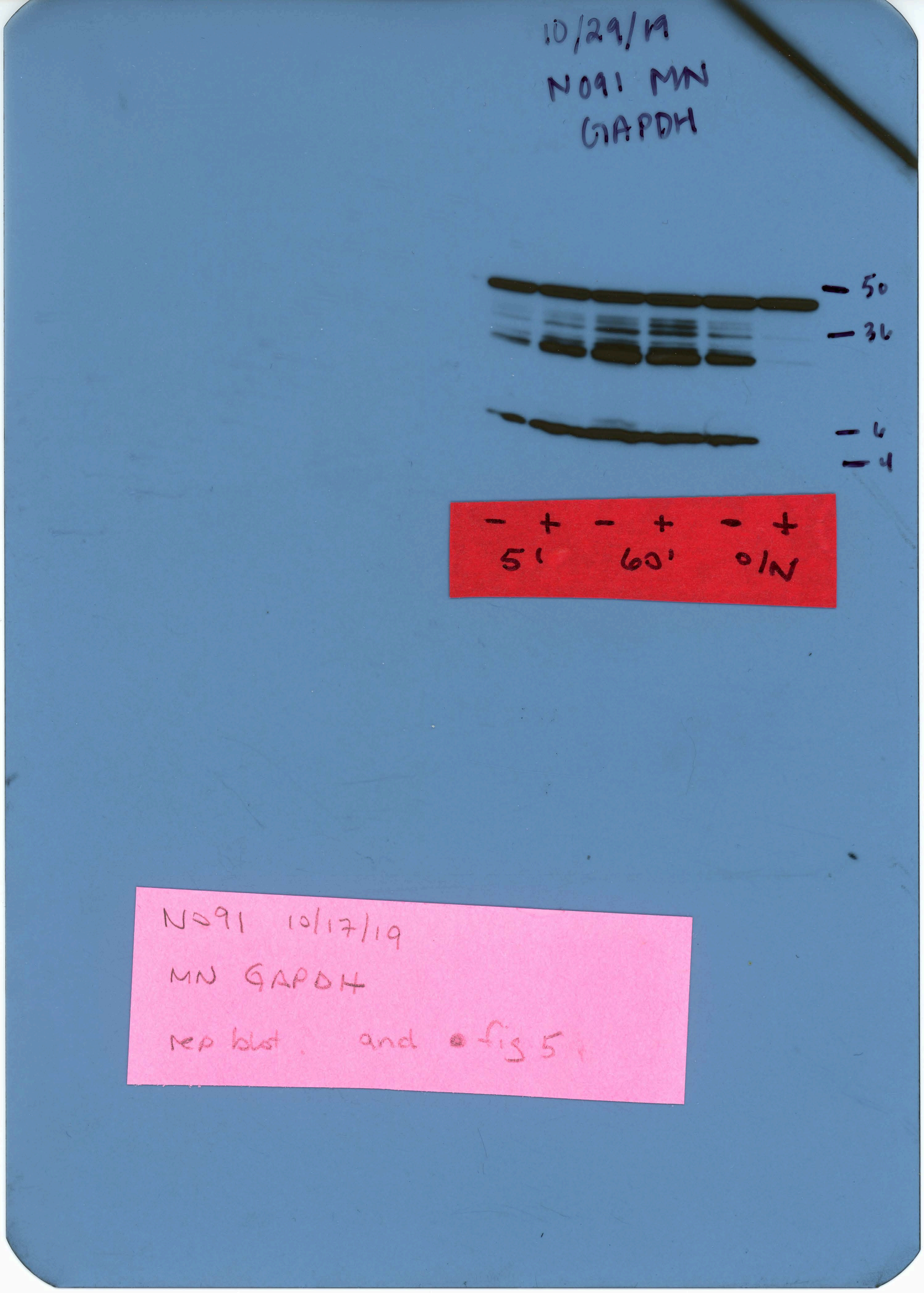
10/29/19
N091 MN
GAPDH
50
36
6
4
− + − + − +
5' 60' O/N
N091 10/17/19
MN GAPDH
rep blot and fig 5

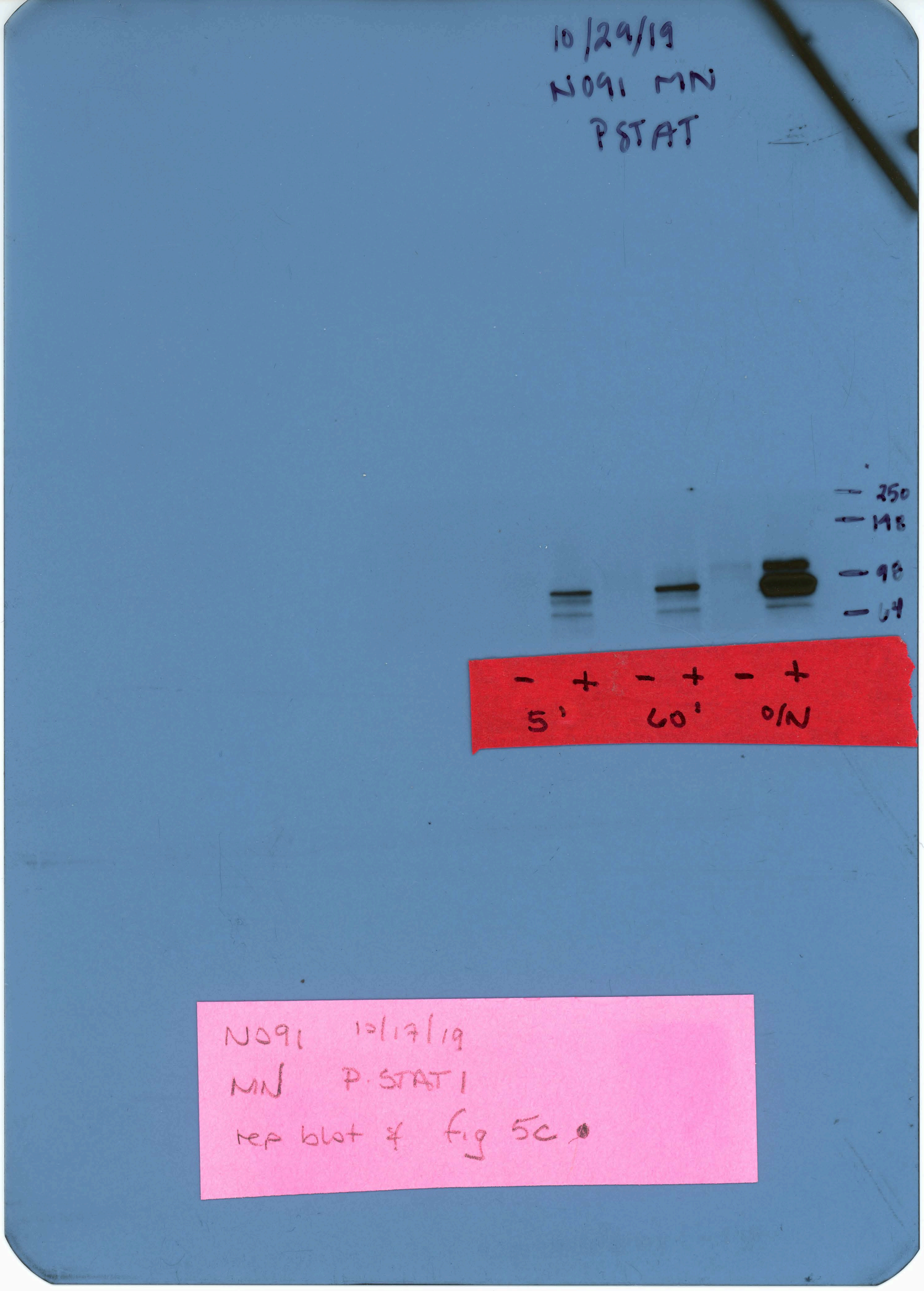

10/29/19
N091 MN
PSTAT
250
148
98
64
- + - + - +
5' 60' O/N
N091 10/17/19
MN P-STAT 1
rep blot of fig 5c

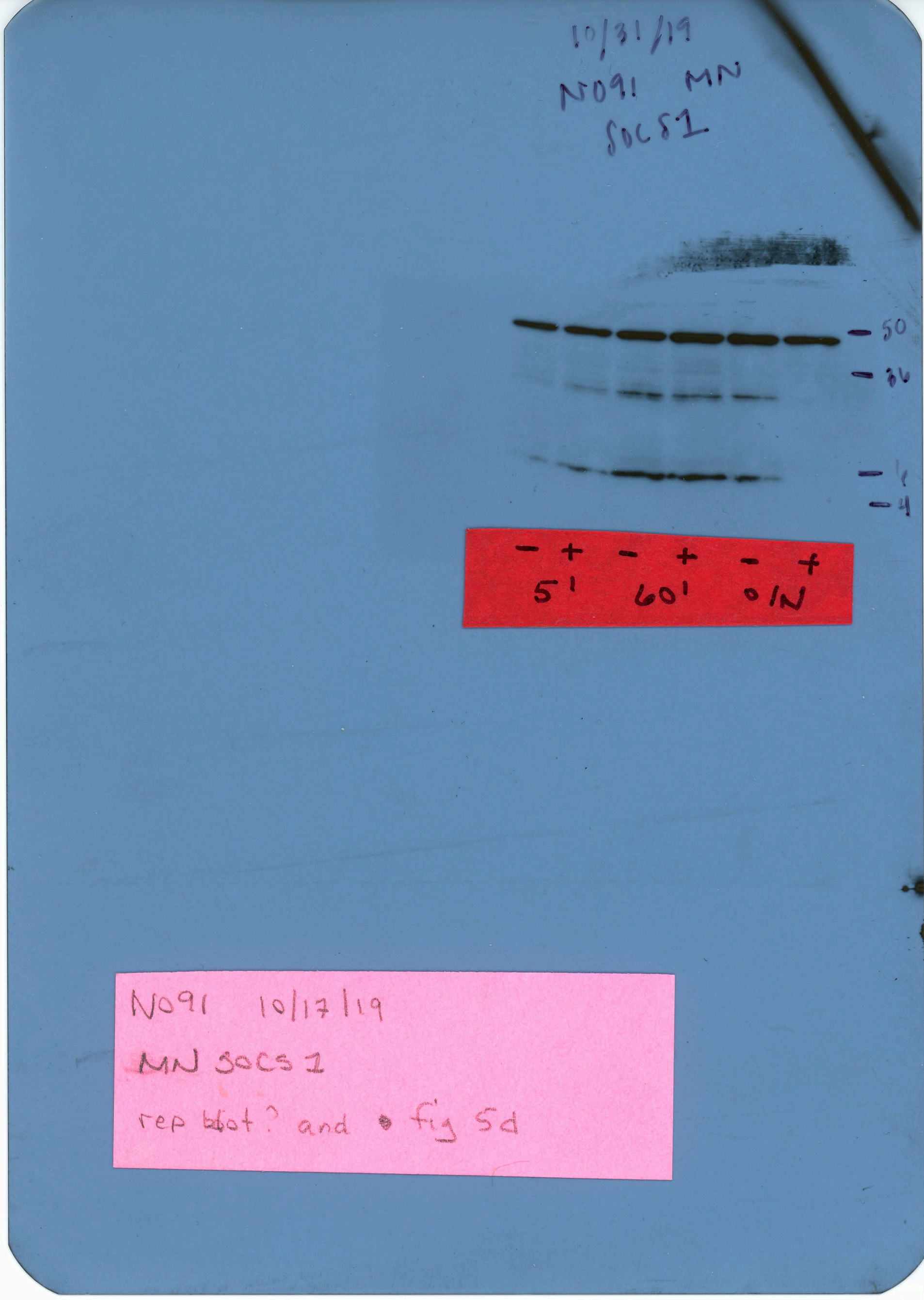
10/31/19
N091 MN
SOCS1
50
− + − + − +
5' 60' O/N
N091 10/17/19
MN SOCS 1
rep blot? and fig 5d

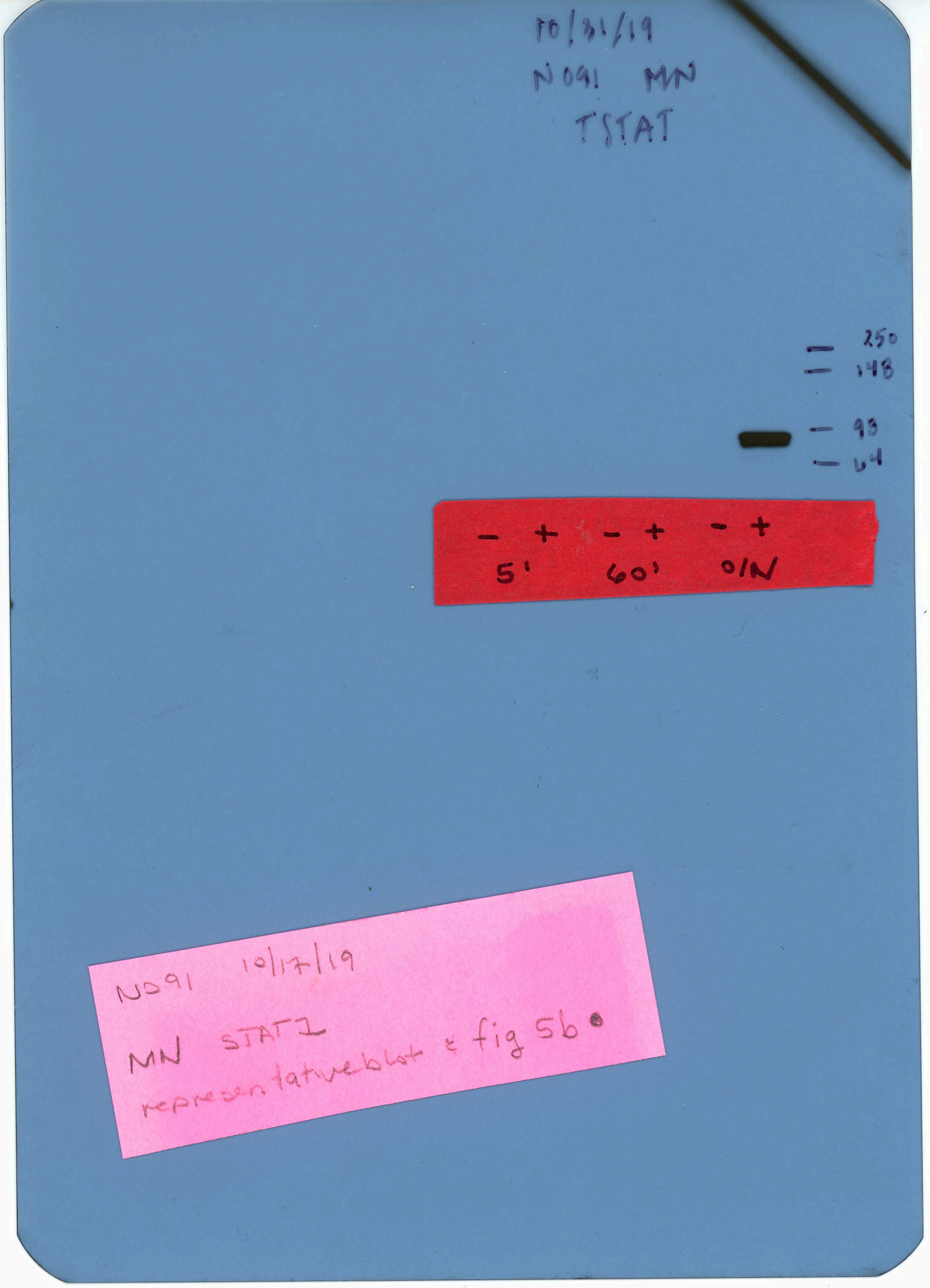
10/31/19
N091 MN
TSTAT
250
148
98
64
− + − + − +
5' 60' O/N
N091 10/17/19
MN STAT1
representative blot & fig 5b

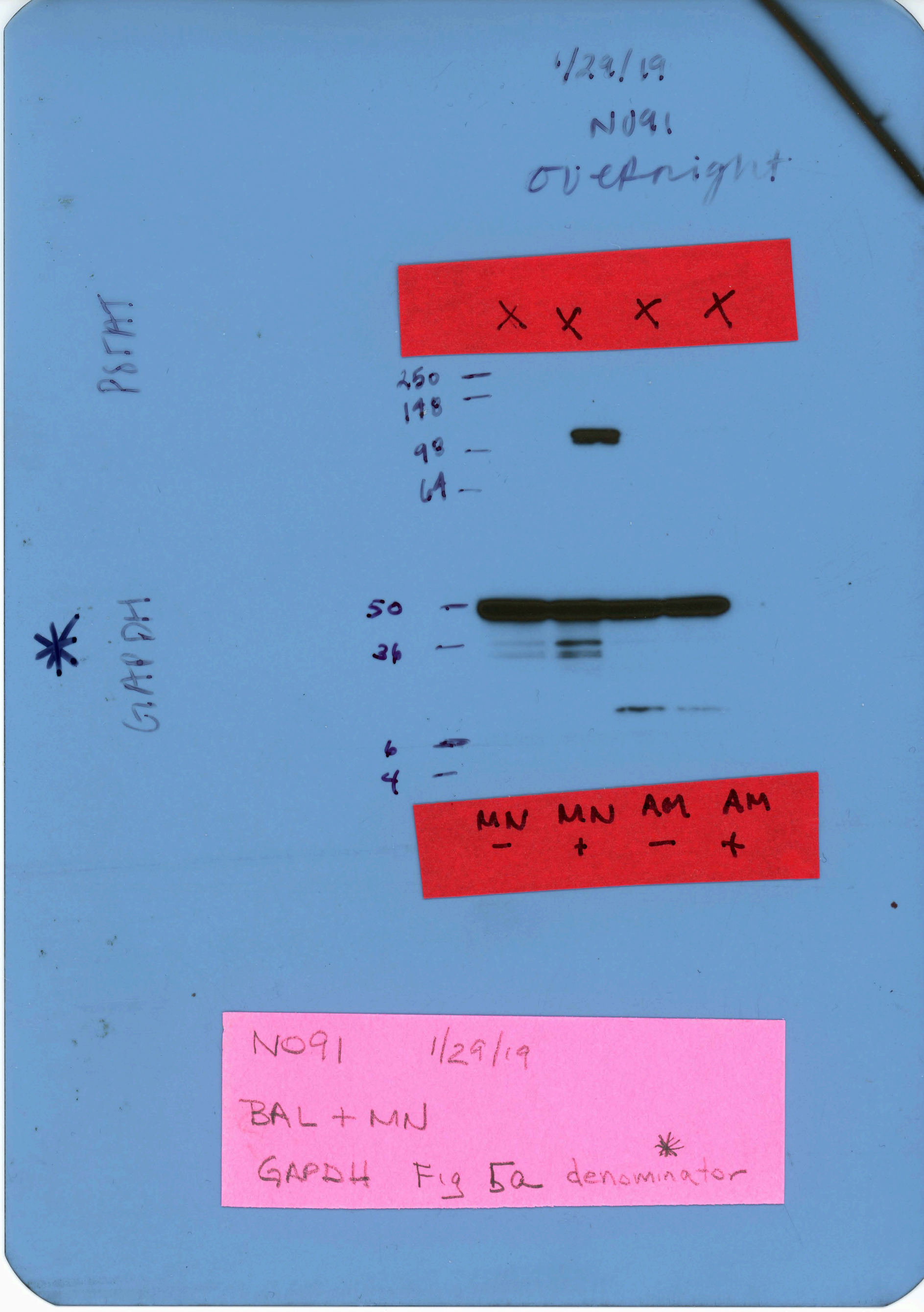
1/29/19
N091
overnight
PSTAT
GAPDH
250
148
98
64
50
36
6
4
MN −
MN +
AM −
AM +
N091 1/29/19
BAL + MN
GAPDH Fig 5a denominator

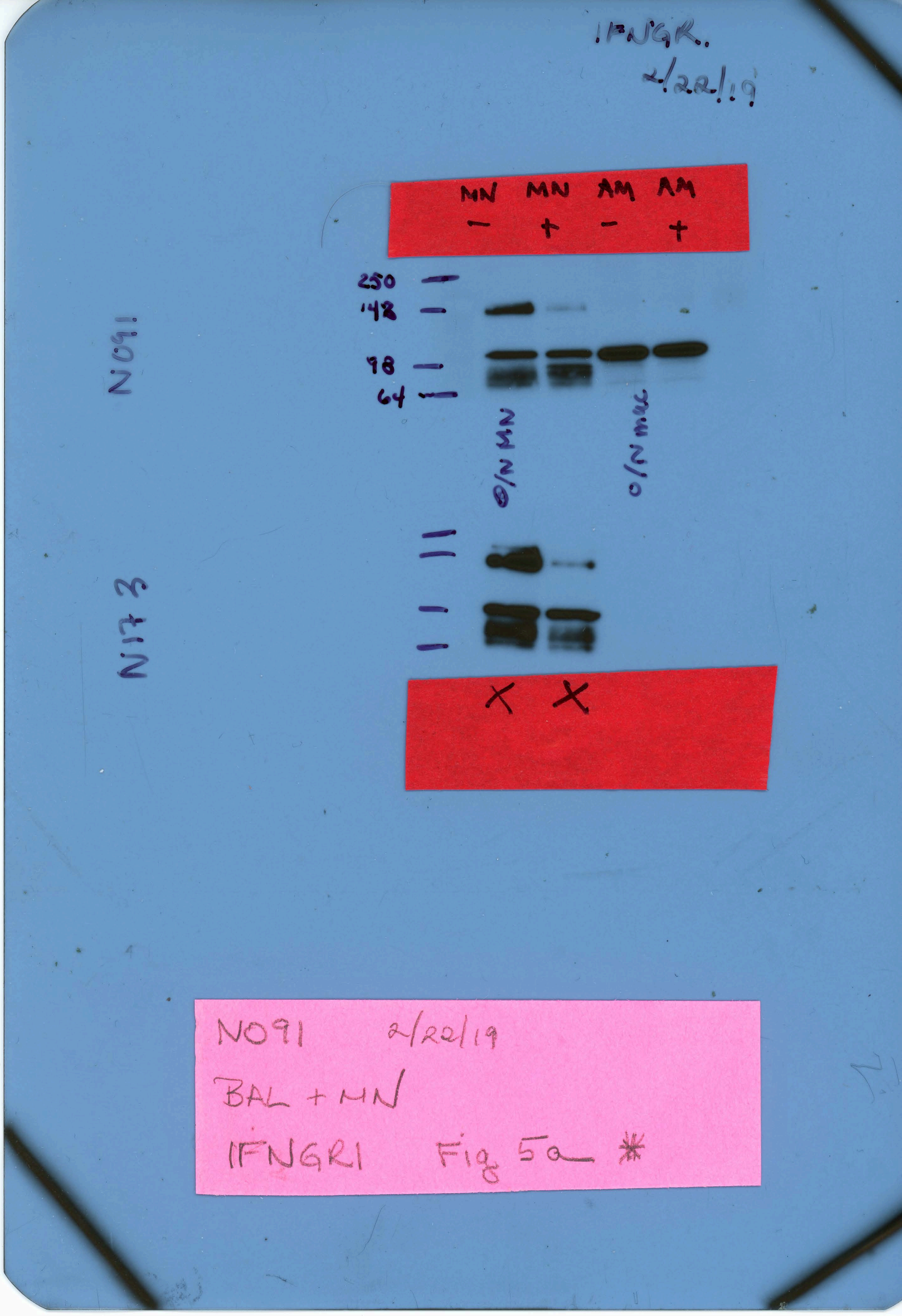
IFNGR.
2/22/19
MN MN AM AM
− + − +
250
148
98
64
N091
N173
O/N MN
N091 2/22/19
BAL + MN
IFNGR1 Fig 5a *

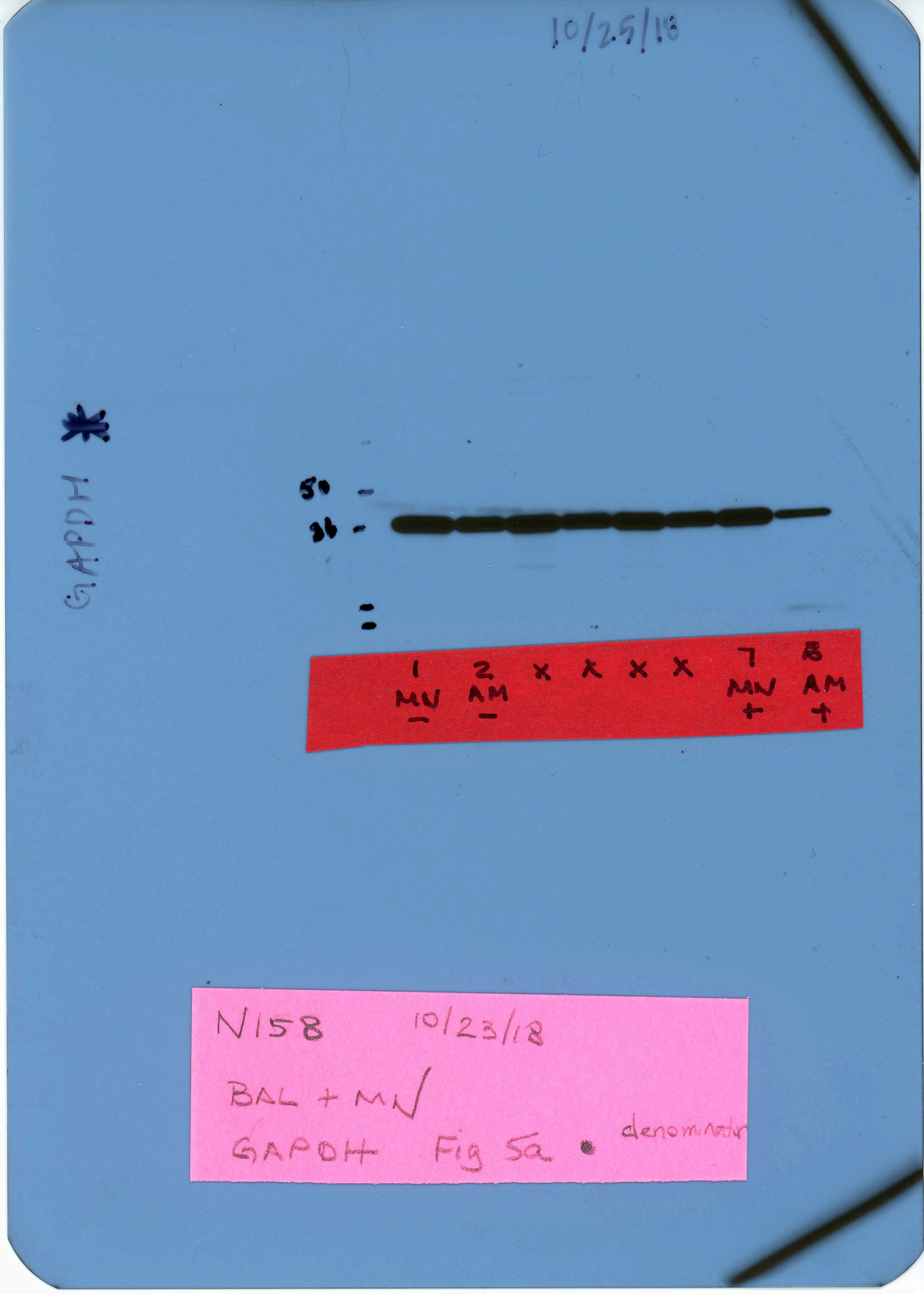
10/25/18
GAPDH
50
36
1 MN –
2 AM –
x x x x
7 MN +
8 AM +
N158 10/23/18
BAL + MN
GAPDH Fig 5a
denominator

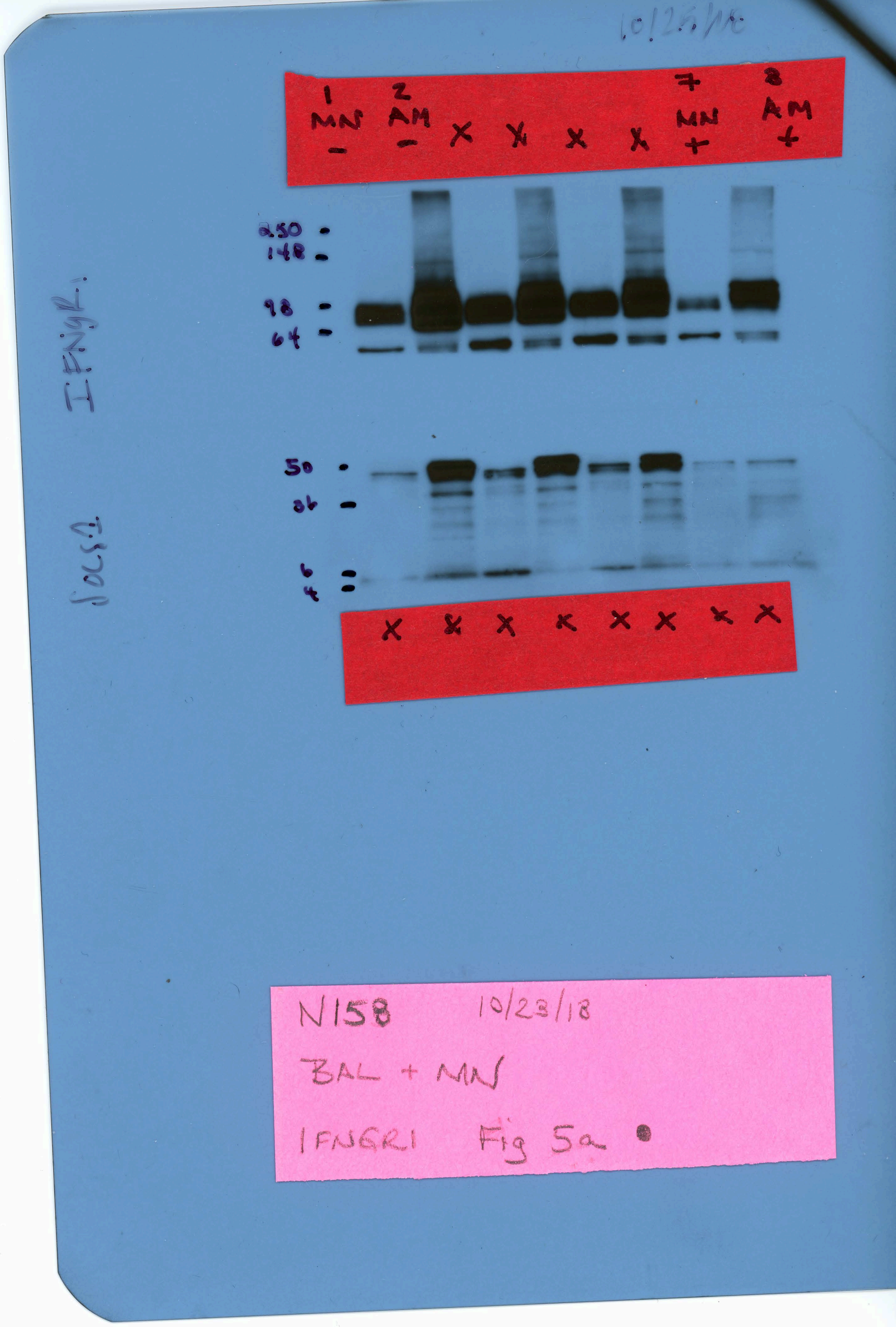
10/23/18
1 MN -
2 AM -
7 MN +
8 AM +
250
148
98
64
IFNgR
50
6
4
N158 10/23/18
BAL + MN
IFNGR1 Fig 5a

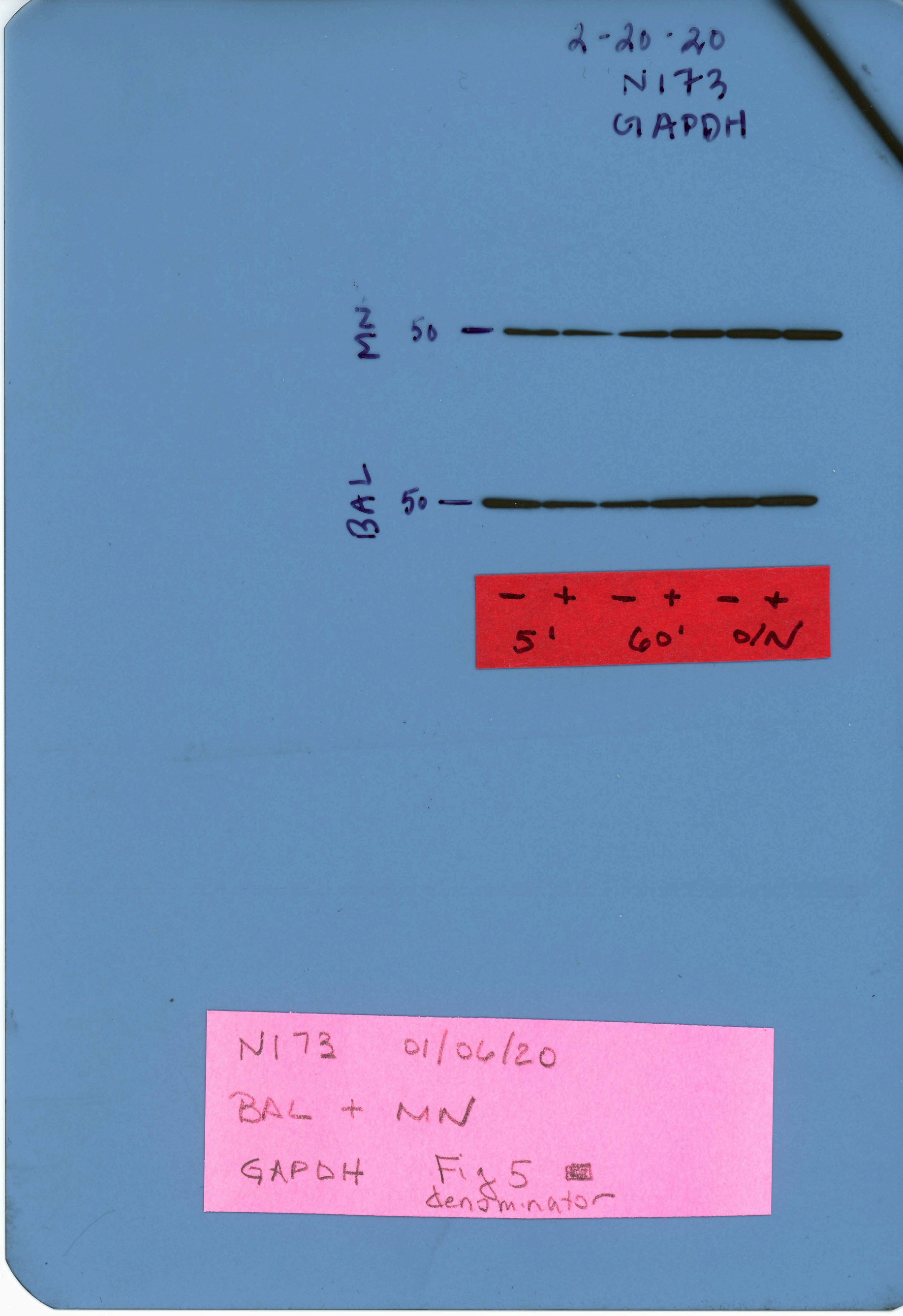

2-20-20
N173
GAPDH
MN
50
BAL
50
- + - + - +
5' 60' O/N
N173 01/06/20
BAL + MN
GAPDH Fig 5
denominator

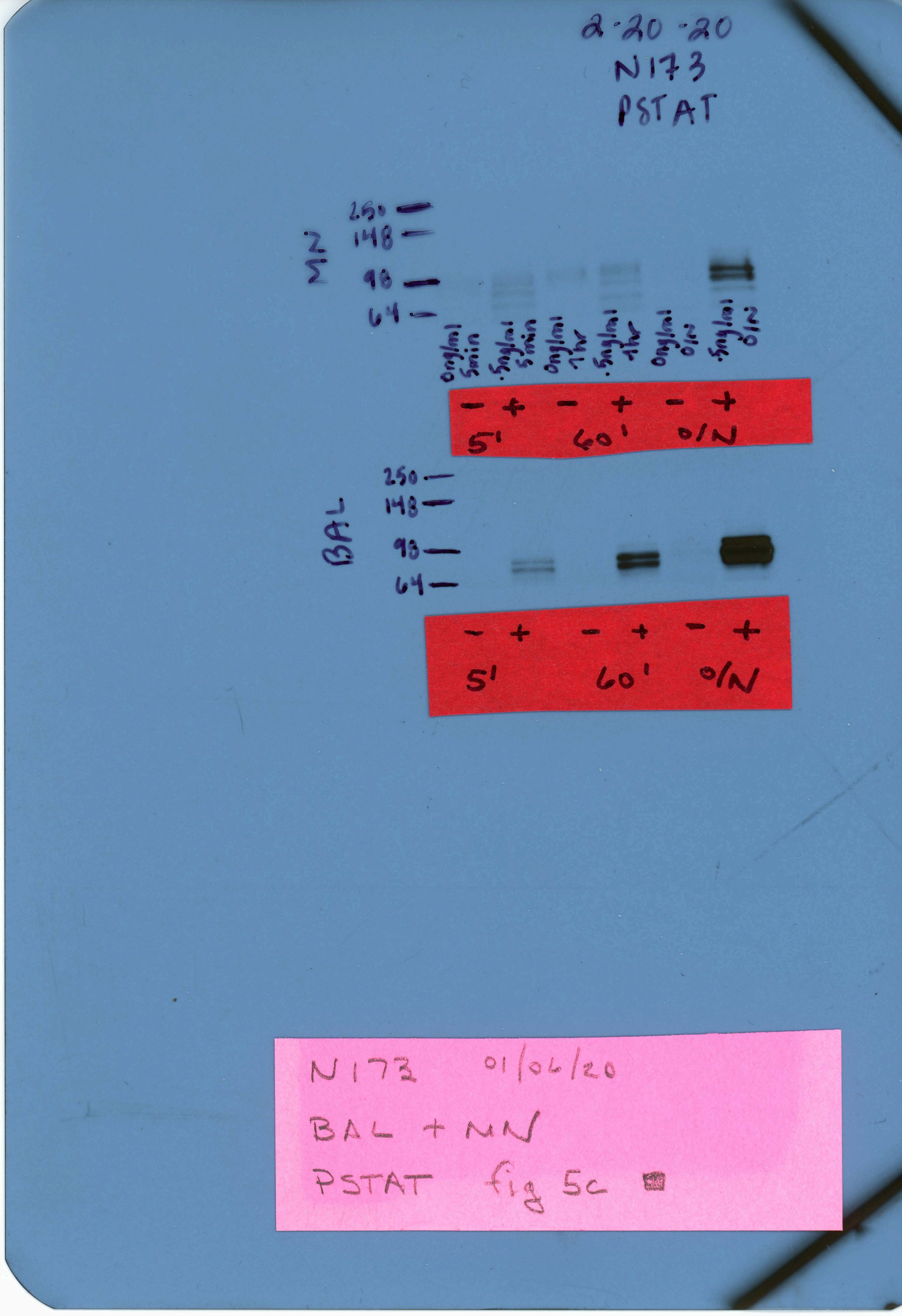
2-20-20
N173
PSTAT
MN
250
148
98
64
0ng/ml 5min
.5ng/ml 5min
0ng/ml 1hr
.5ng/ml 1hr
0ng/ml O/N
.5ng/ml O/N
− + − + − +
5' 60' O/N
BAL
250
148
98
64
− + − + − +
5' 60' O/N
N173 01/06/20
BAL + MN
PSTAT fig 5c

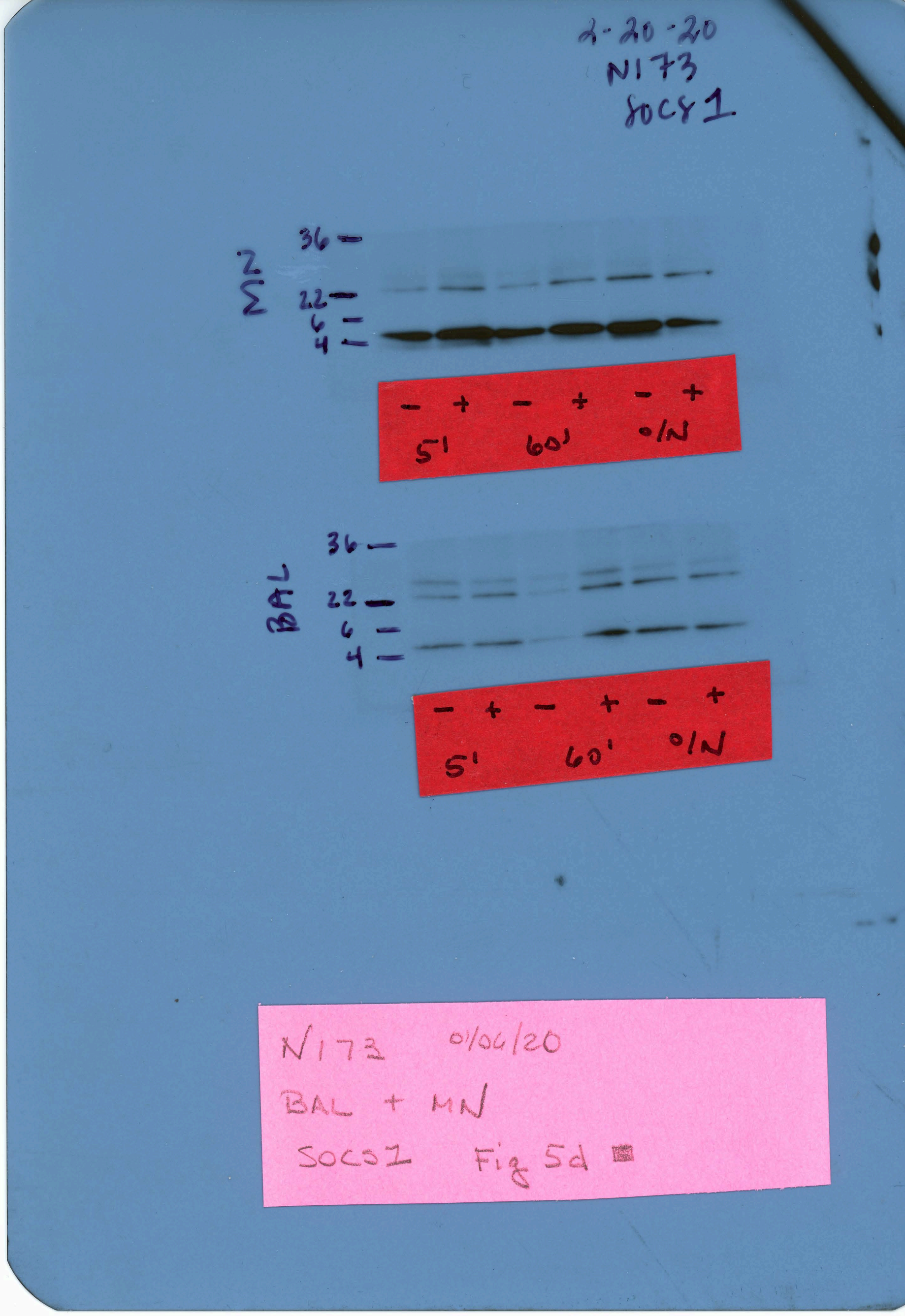
2-20-20
N173
SOCS1
MN
36
22
6
4
- + - + - +
5' 60' O/N
BAL
36
22
6
4
- + - + - +
5' 60' O/N
N173 01/06/20
BAL + MN
SOCS1 Fig 5d

2-21-20
N173
TSTAT

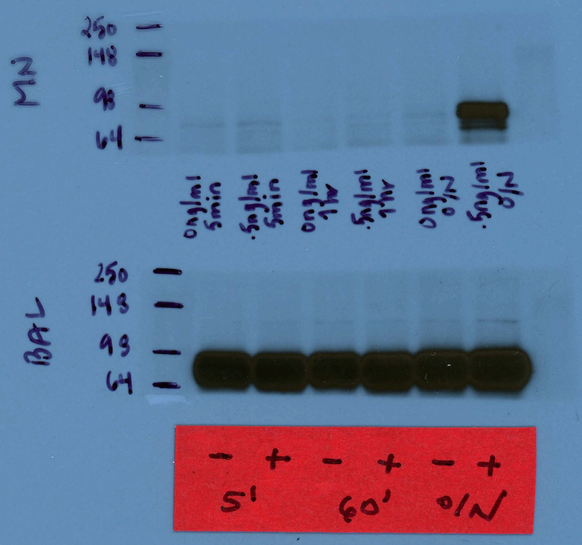

N173 01/06/20
BAL + MN
STAT 1 Fig 5b

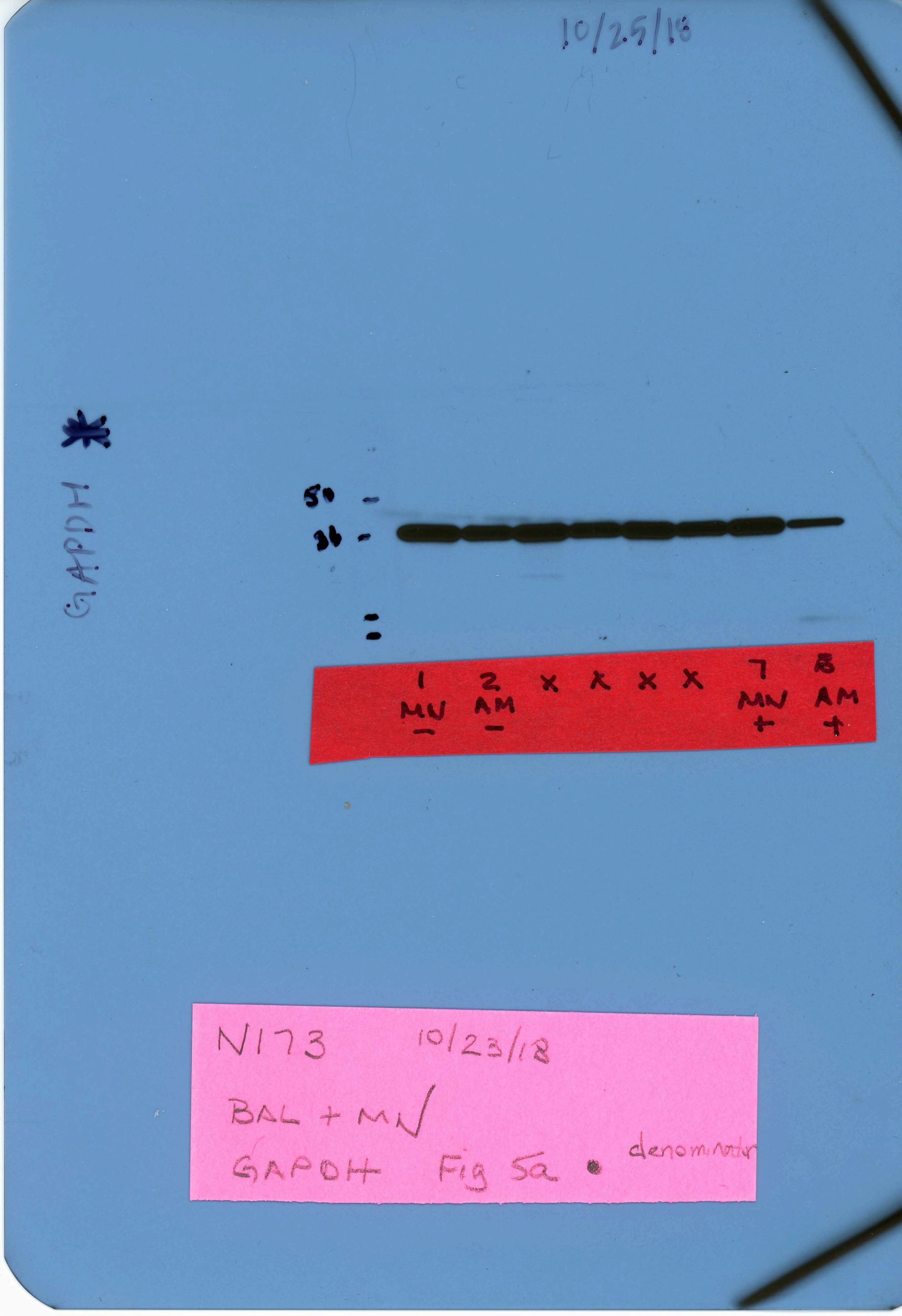

10/25/18
GAPDH
50
36
1 MN –
2 AM –
x x x x
7 MN +
8 AM +
N173 10/23/18
BAL + MN
GAPDH Fig 5a • denominator

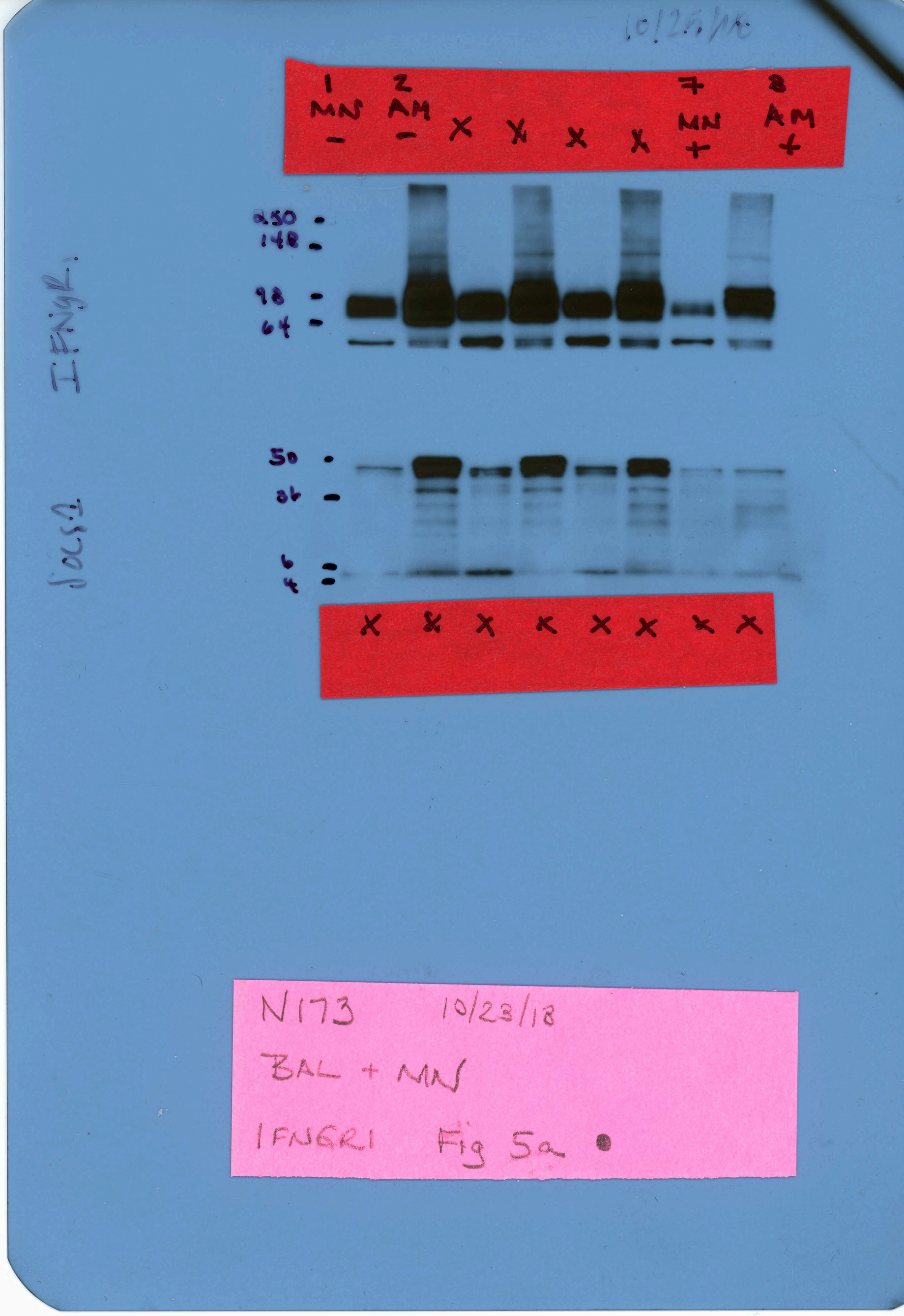

1 MN −
2 AM −
7 MN +
8 AM +
250
148
98
64
50
36
6
4
N173 10/23/18
BAL + MN
IFNGR1 Fig 5a

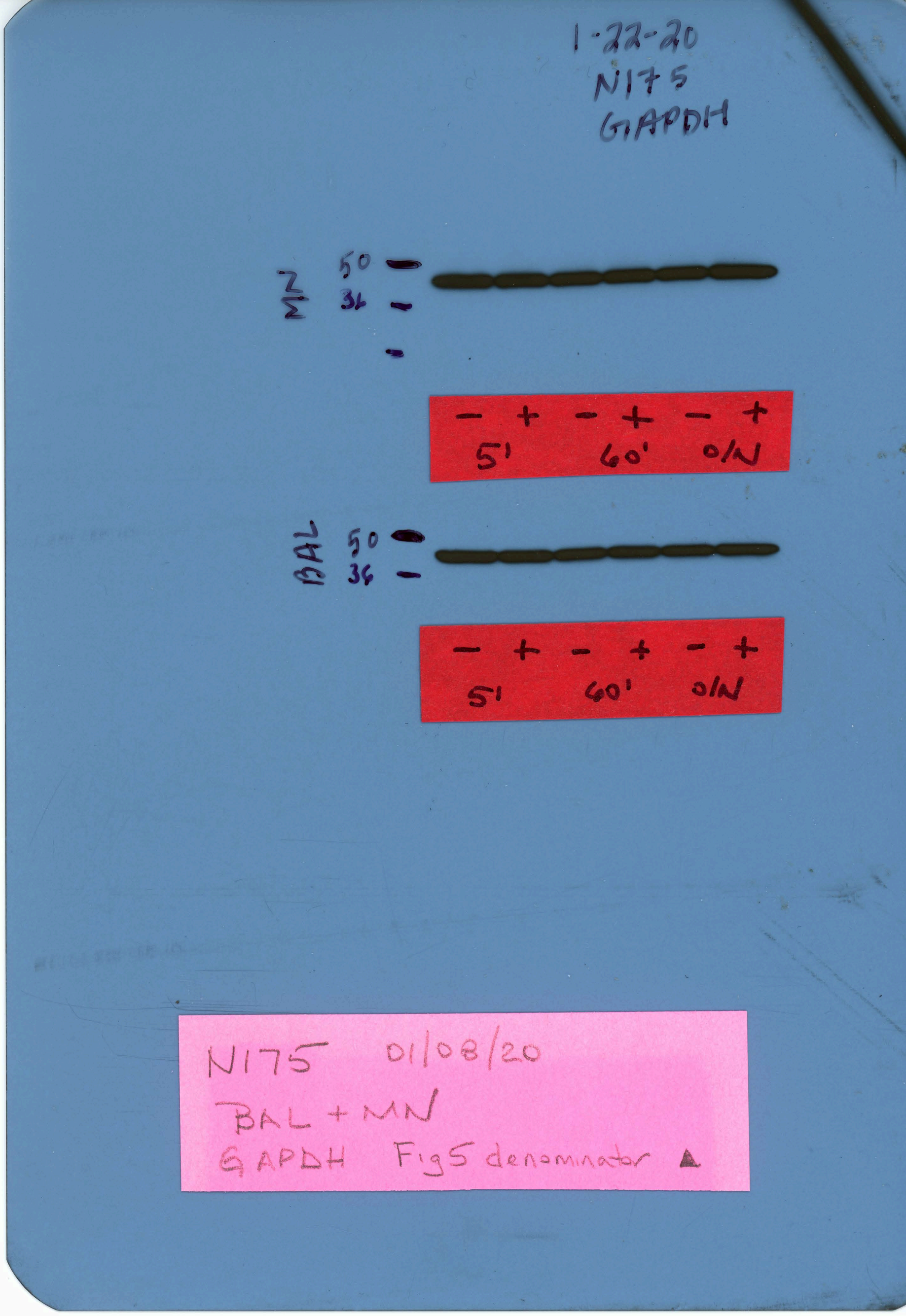

1-22-20
N175
GAPDH
MN
50
36
− + − + − +
5' 60' O/N
BAL
50
36
− + − + − +
5' 60' O/N
N175 01/08/20
BAL + MN
GAPDH Fig5 denominator ▲

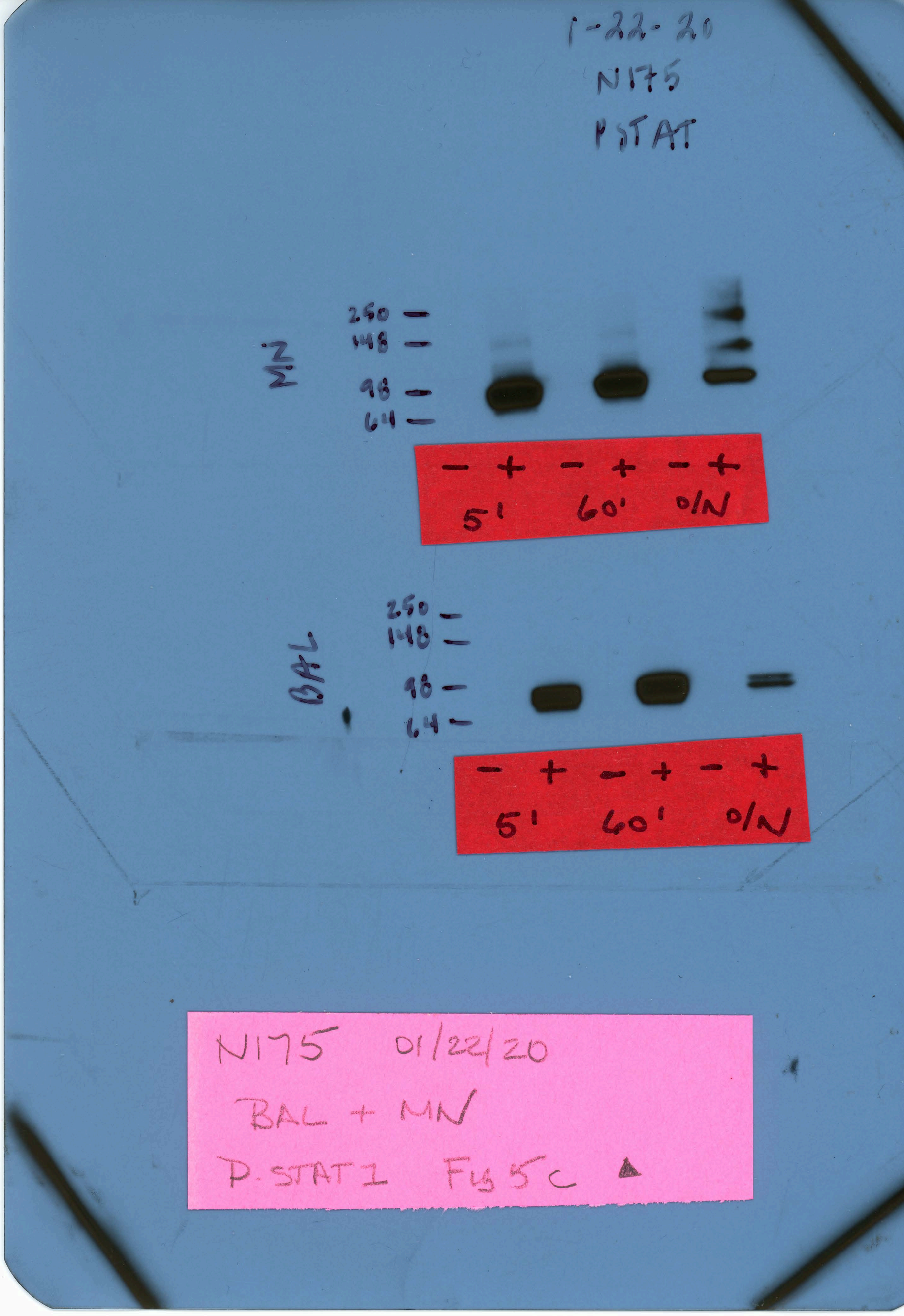
1-22-20
N175
PSTAT
MN
250
148
98
64
− + − + − +
5' 60' O/N
BAL
250
148
98
64
− + − + − +
5' 60' O/N
N175 01/22/20
BAL + MN
P.STAT1 Fig 5C

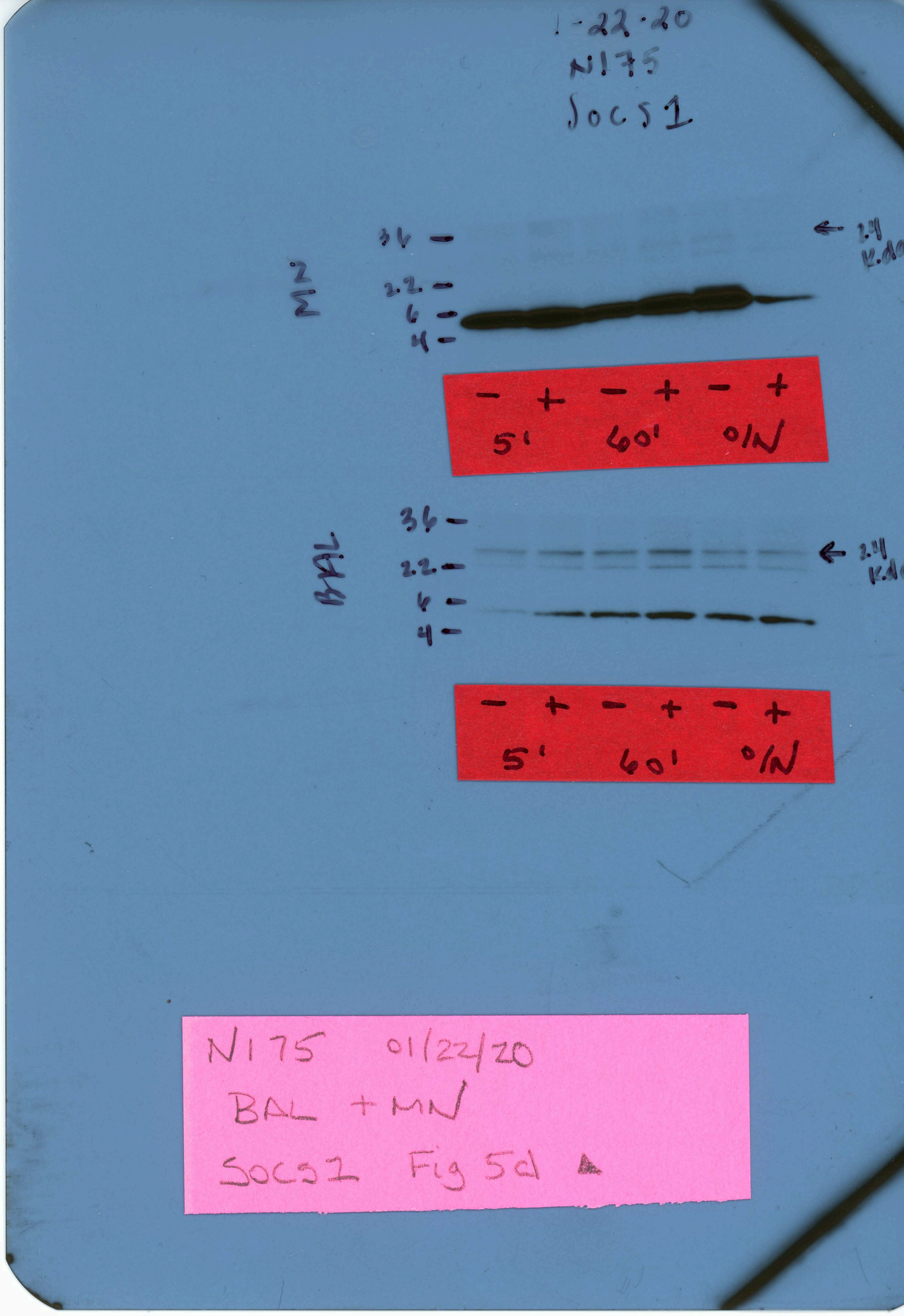
1-22-20
N175
Socs1
MN
36 -
22 -
6 -
4 -
- + - + - +
5' 60' O/N
BAL
36 -
22 -
6 -
4 -
- + - + - +
5' 60' O/N
N175 01/22/20
BAL + MN
Socs1 Fig 5d

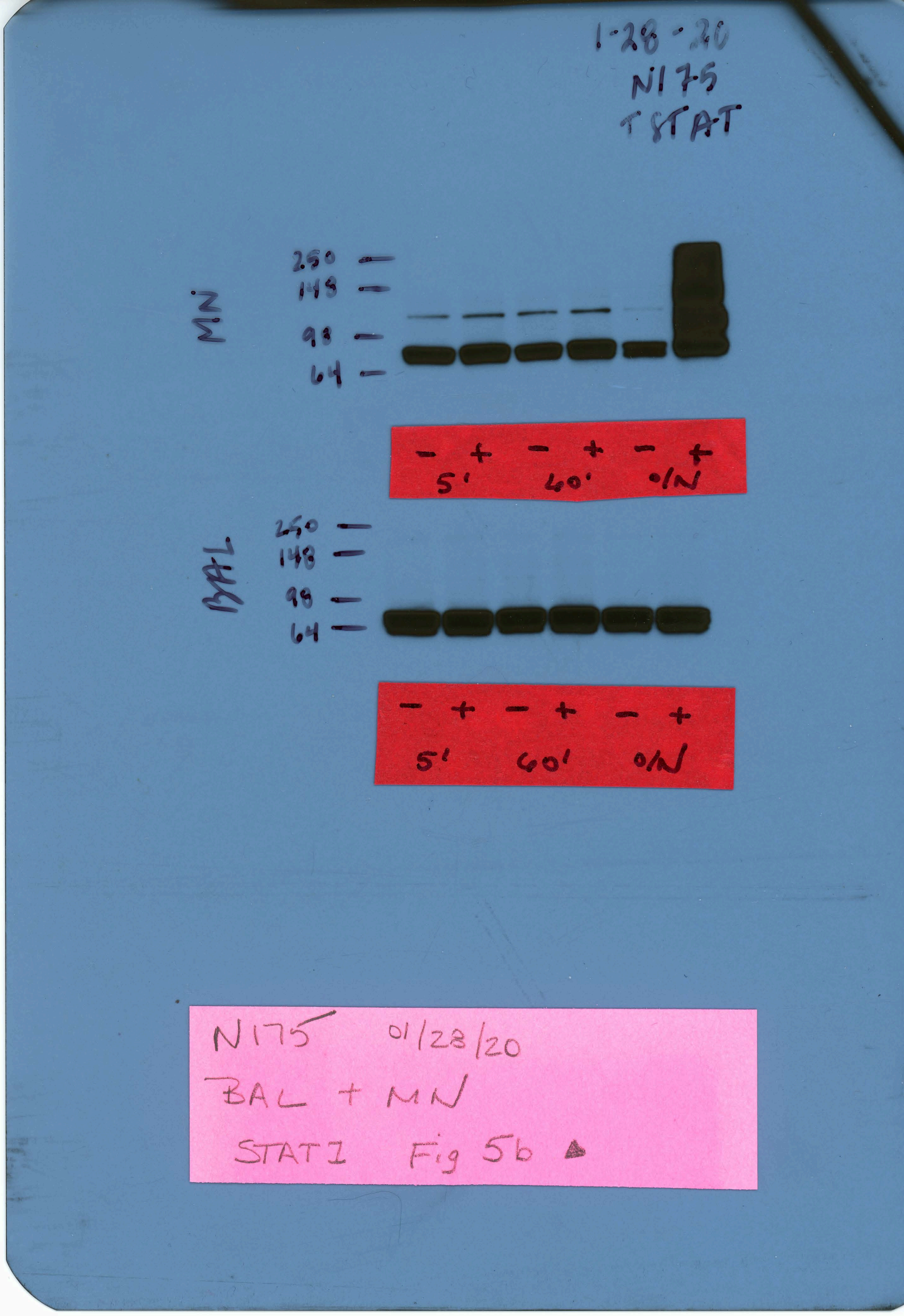
1-28-20
N175
T STAT
MN
250
148
98
64
− + − + − +
5' 60' O/N
BAL
250
148
98
64
− + − + − +
5' 60' O/N
N175 01/28/20
BAL + MN
STAT1 Fig 5b

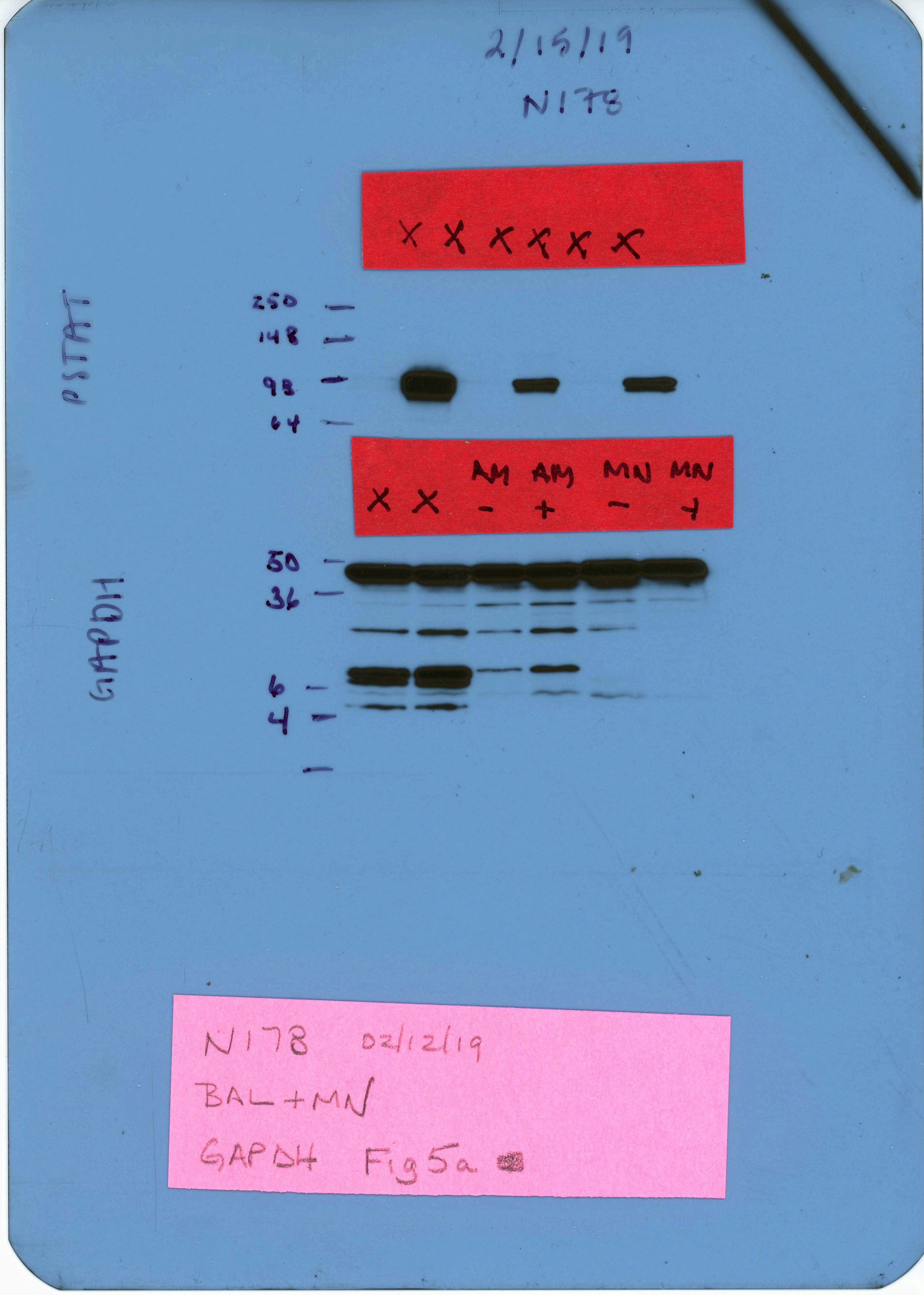
2/15/19
N178
X X X X X X
250
148
98
64
pSTAT
AM AM MN MN
X X - + - +
50
36
6
4
GAPDH
N178 02/12/19
BAL+MN
GAPDH Fig5a

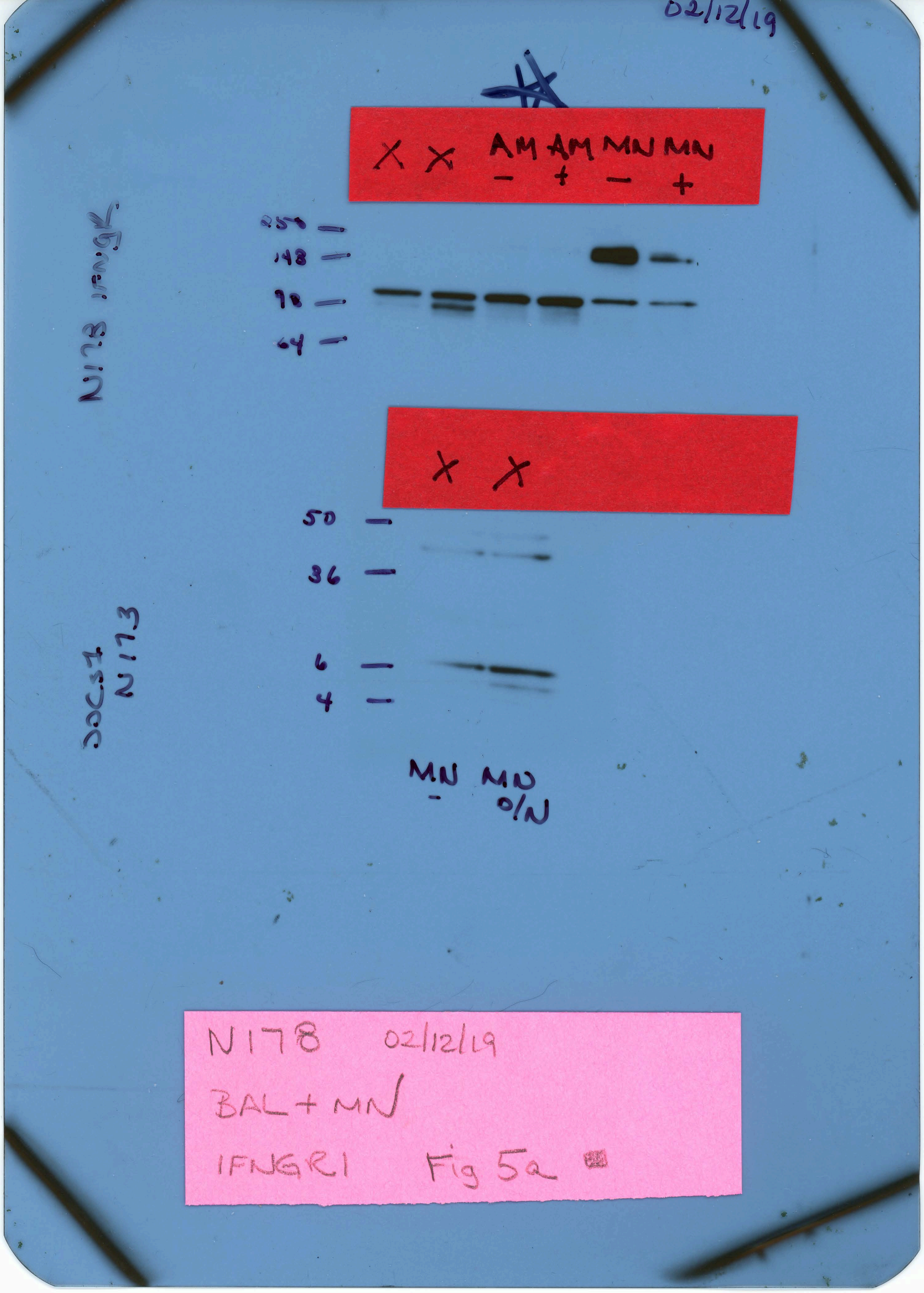
02/12/19
X X AM AM MN MN
– + – +
148
98
64
50
36
6
4
MN –
MN O/N
N178 02/12/19
BAL + MN
IFNGR1 Fig 5a

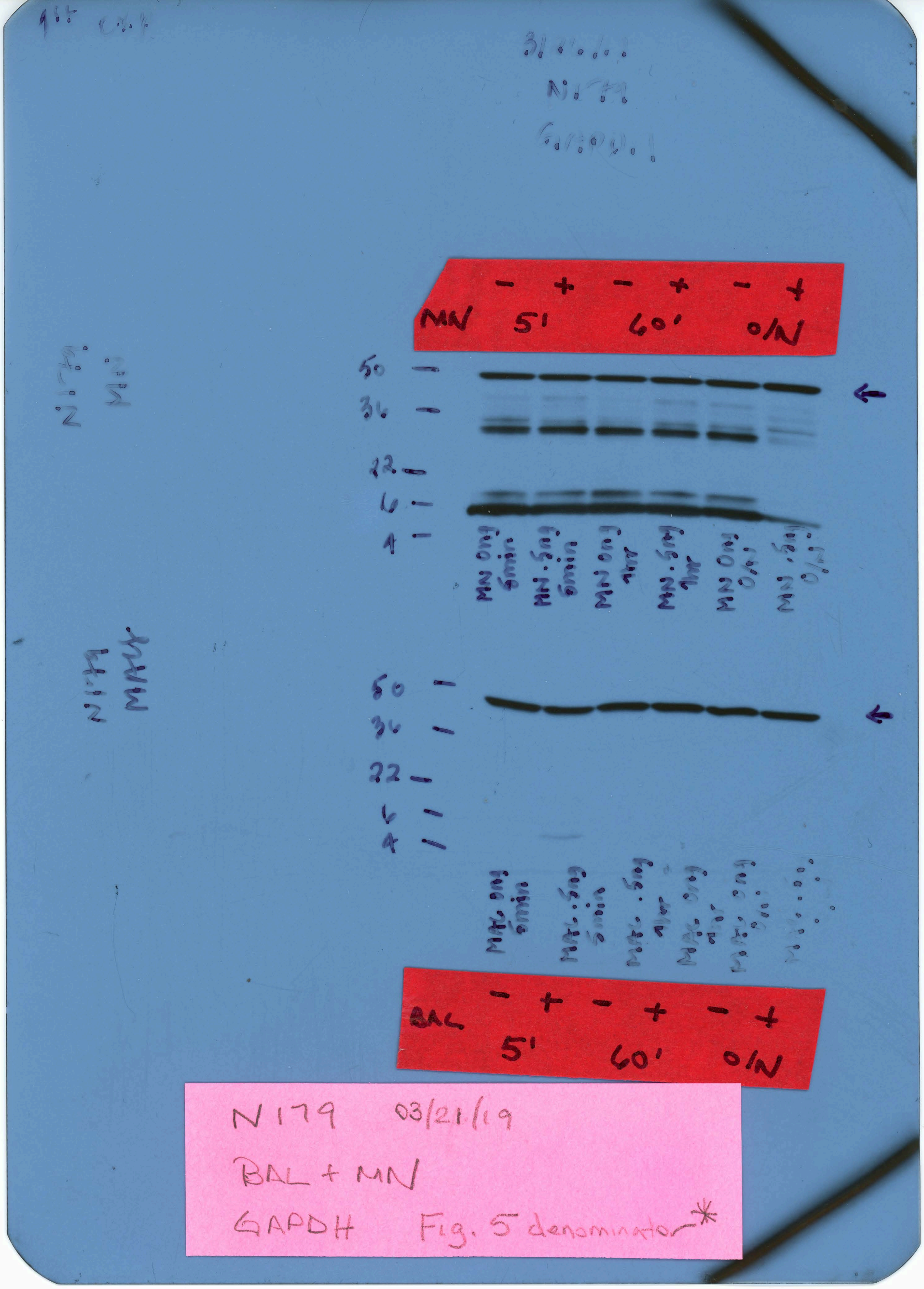
– + – + – +
MN 5' 60' O/N
50
36
22
6
4
– + – + – +
BAL 5' 60' O/N
N179 03/21/19
BAL + MN
GAPDH Fig. 5 denominator*

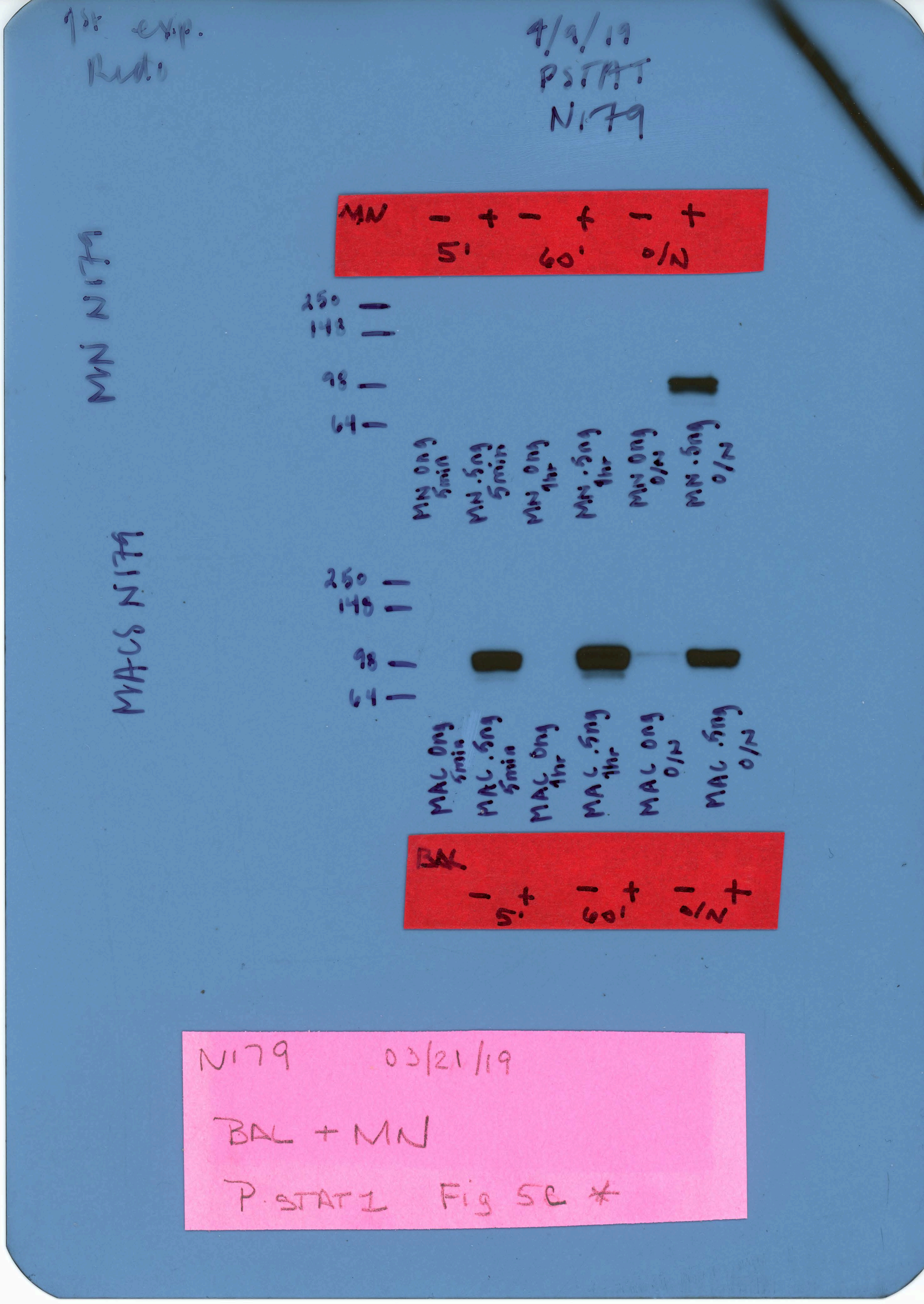
4/9/19
PSTAT
N179
MN N179
MN - + - + - +
5' 60' O/N
250
148
98
64
MACS N179
BAL - + - + - +
5' 60' O/N
N179 03/21/19
BAL + MN
P STAT 1 Fig 5C *

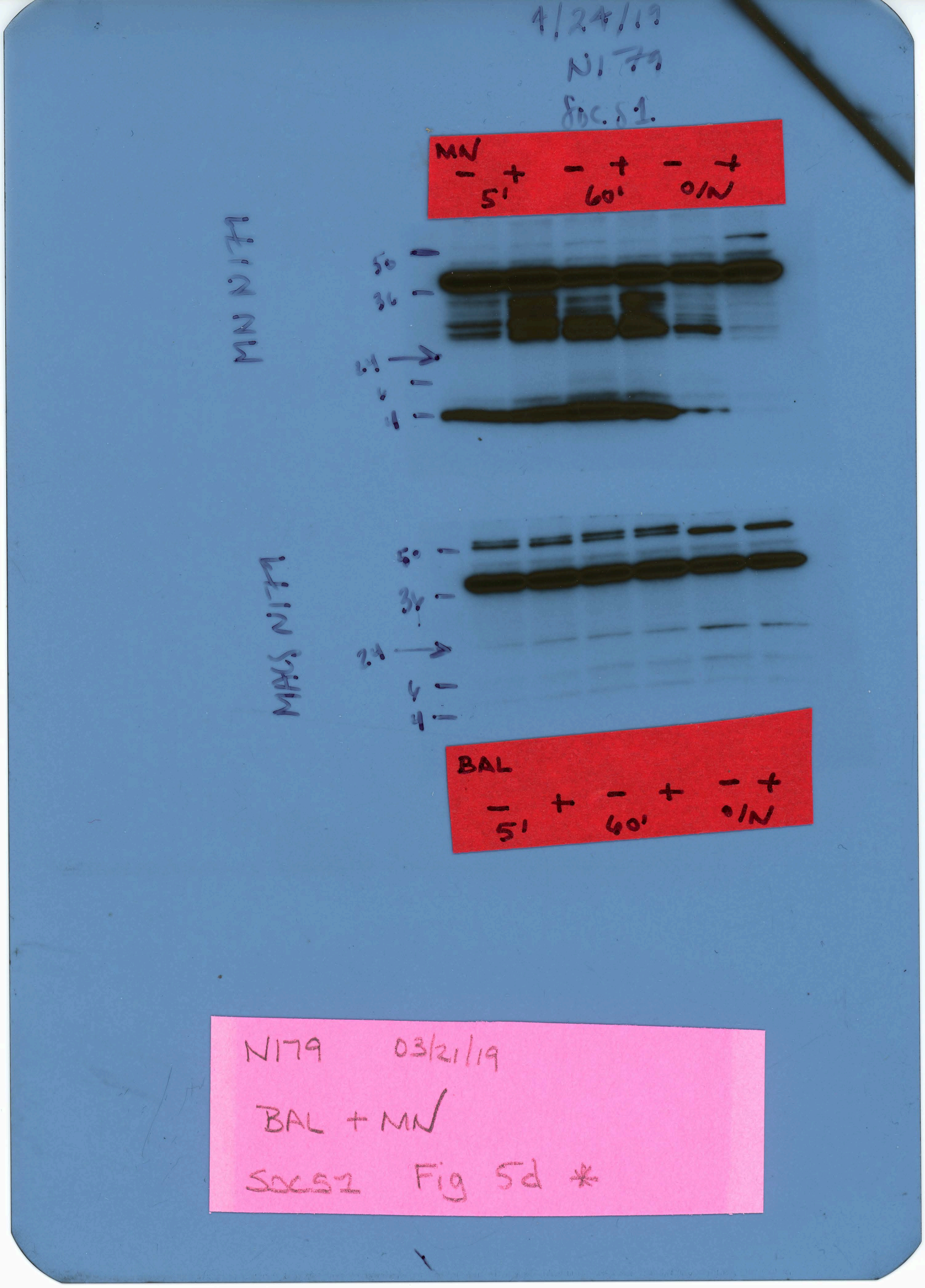

N179
MN
- + - + - +
5' 60' O/N
MN N179
BAL
- + - + - +
5' 60' O/N
N179 03/21/19
BAL + MN
Fig 5d *

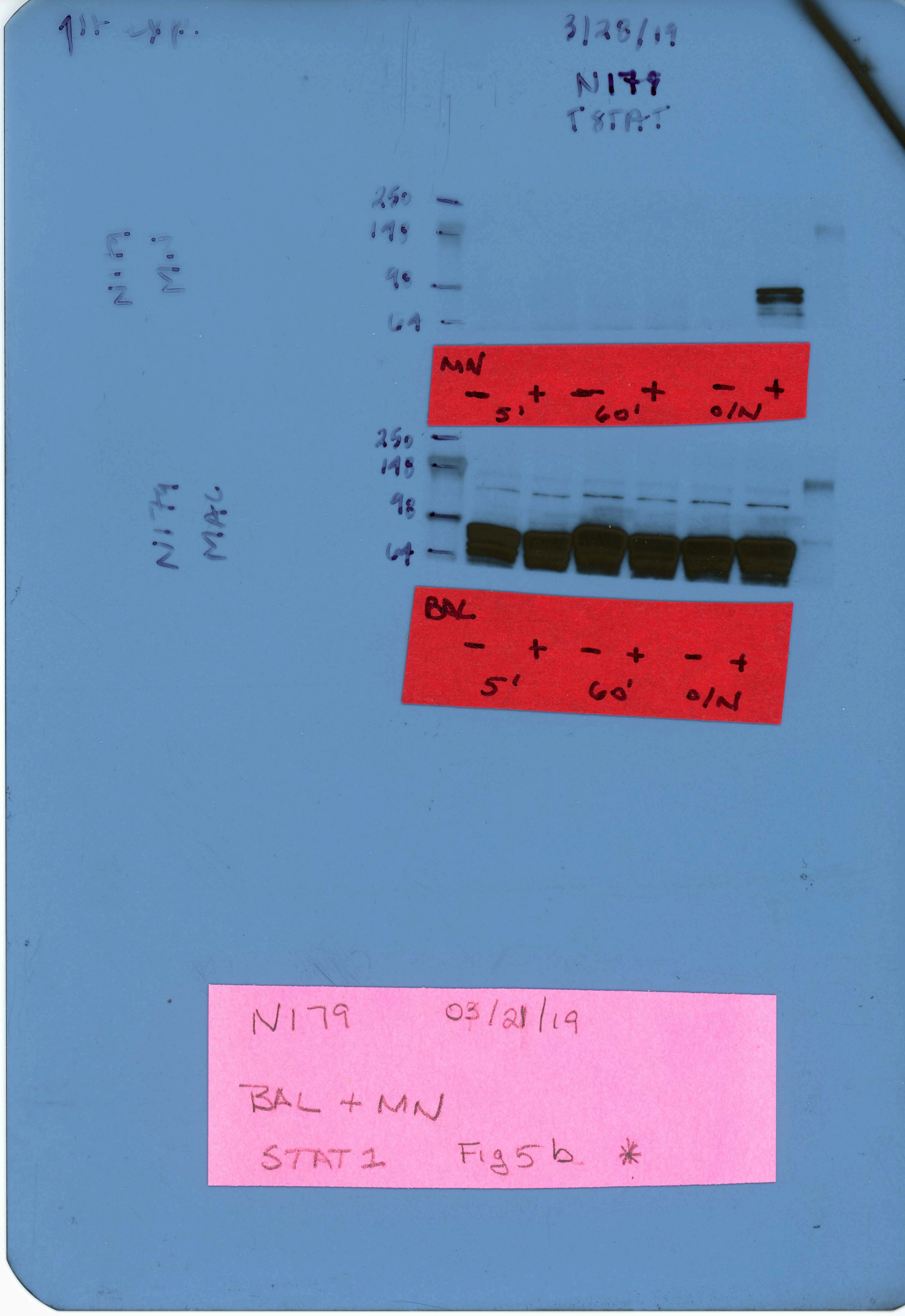

3/28/19
N179
MN
- 5' + - 60' + - O/N +
BAL
- + - + - +
5' 60' O/N
N179 03/21/19
BAL + MN
STAT1 Fig5b *
